# *C9orf72* ablation causes immune dysregulation characterized by leukocyte expansion, autoantibody production, and glomerulonephropathy in mice

**DOI:** 10.1038/srep23204

**Published:** 2016-03-16

**Authors:** Amanda Atanasio, Vilma Decman, Derek White, Meg Ramos, Burcin Ikiz, Hoi-Ching Lee, Chia-Jen Siao, Susannah Brydges, Elizabeth LaRosa, Yu Bai, Wen Fury, Patricia Burfeind, Ralica Zamfirova, Gregg Warshaw, Jamie Orengo, Adelekan Oyejide, Michael Fralish, Wojtek Auerbach, William Poueymirou, Jan Freudenberg, Guochun Gong, Brian Zambrowicz, David Valenzuela, George Yancopoulos, Andrew Murphy, Gavin Thurston, Ka-Man Venus Lai

**Affiliations:** 1 Regeneron Pharmaceuticals, Inc, Tarrytown, NY USA

## Abstract

The expansion of a hexanucleotide (GGGGCC) repeat in *C9ORF72* is the most common cause of amyotrophic lateral sclerosis (ALS) and frontotemporal dementia (FTD). Both the function of C9ORF72 and the mechanism by which the repeat expansion drives neuropathology are unknown. To examine whether C9ORF72 haploinsufficiency induces neurological disease, we created a C9orf72-deficient mouse line. Null mice developed a robust immune phenotype characterized by myeloid expansion, T cell activation, and increased plasma cells. Mice also presented with elevated autoantibodies and evidence of immune-mediated glomerulonephropathy. Collectively, our data suggest that C9orf72 regulates immune homeostasis and an autoimmune response reminiscent of systemic lupus erythematosus (SLE) occurs in its absence. We further imply that haploinsufficiency is unlikely to be the causative factor in C9ALS/FTD pathology.

The *C9ORF72* locus has been researched extensively following identification of an expanded hexanucleotide (GGGGCC) repeat in *C9ORF72* as the most common cause of sporadic and familial forms of amyotrophic lateral sclerosis (ALS) and frontotemporal dementia (FTD)^1^,^2^. FTD is characterized by cognitive and behavioral symptoms and ALS by motor neuron degeneration, yet extensive genetic, clinical, and neuropathological overlap indicate the two conditions form opposite ends of a continuous disease spectrum^3^. Patients may develop ALS, FTD, or both (C9ALS/FTD) and generally carry one normal allele comprised of 2–16 copies of the repeat and an expanded pathogenic allele with repeats numbering in the hundreds to thousands.

The *C9ORF72* repeat is intronic^1^,^2^, therefore the mechanism by which the repeat expansion causes neuronal cell death is unclear. Toxic buildup of unspliced, repetitive mRNAs is one theory. Studies have demonstrated that *C9ORF72* repeats sequester certain RNA binding proteins into cytoplasmic foci, perhaps reducing or preventing protein synthesis needed for normal cellular processes^4^,^5^,^6^,^7^,^8^,^9^,^10^. An alternate hypothesis implicates insoluble dipeptide chains arising from Repeat-Associated non-ATG (RAN) translation of the repeats. C9ALS/FTD autopsy brain sections contain cytoplasmic poly-glycine-proline peptide inclusions^7^,^11^,^12^,^13^,^14^ that could cause neurotoxicity in a manner similar to the neurofibrillary tangles and amyloid plaques of Alzheimer’s disease^15^. Both theories cast the repeat as a gain-of-function lesion that may or may not impact the function of *C9ORF72* itself.

A third theory to explain C9ALS/FTD pathogenicity is haploinsufficiency of *C9ORF72*. Repeat expansion as a loss-of-function mutation is suggested by studies on repeat carriers who express roughly half as much *C9ORF72* transcript as individuals with two unexpanded copies^16^,^17^. In addition, the repeat can cause DNA and RNA to form four-stranded G-quadruplexes. Poor transcription/translation of quadruplexed DNA/RNA also implicate haploinsufficiency, and therefore impaired *C9ORF72* function as pathogenic^18^,^19^,^20^.

Functional studies in *C. elegans* and zebrafish support the haploinsufficiency hypothesis by demonstrating that a reduction in C9ORF72 homolog levels results in locomotion defects^21^,^22^. However, mouse studies suggest otherwise. Conditional *C9orf72* ablation in neurons and glial cells or intracerebral mRNA knockdown did not cause motor neuron disease, gliosis, TDP-43 pathology, or increased ubiquitination, defects associated with C9ALS/FTD^23^,^24^. These results imply haploinsufficiency in the central nervous system (CNS) is not pathogenic; however ablation may not have occurred in a crucial cell type and knockdown could have allowed residual C9orf72 expression. The conflicting results and variability intrinsic to cell-specific gene ablation or message knockdown warrant further study of *C9orf72* in a universal knockout (*C9orf72*^***−/−***^).

In this study, we separate effects intrinsic to the repeat from the function of C9ORF72 by genetically ablating the mouse homolog, *3110043O21Rik* (hereafter referred to as *C9orf72*). Heterozygous (*C9orf72*^+/−^) mice were healthy and lived normal lifespans. In contrast, *C9orf72*^***−/−***^ mice developed an autoimmune phenotype consisting of expansions in myeloid and lymphoid cell populations, autoantibody production, and glomerulonephropathy. Mild, nonspecific neurological deficits arose after the immune response was established but *C9orf72* ablation did not result in classic motor neuron degeneration. Our results indicate haploinsufficiency is not the main cause of C9ALS/FTD neuropathology and describe a novel role for C9ORF72 in immune homeostasis.

## Results

### *C9orf72* ^*−/−*^ mouse engineering and validation

To create a model of C9orf72 haploinsufficiency, we replaced the mouse *C9orf72* coding sequence and introns with a *lacZ* reporter (Supplemental Fig. 1A). To confirm *C9orf72* ablation, we performed gene-specific Taqman analyses on wildtype (WT), *C9orf72*^+/−^ and *C9orf72*^***−/−***^ tissue cDNA. We detected high *C9orf72* expression in WT central nervous system (CNS), fat, and muscle with lower levels in lymphoid tissues. *C9orf72*^+/−^ mice had roughly half the expression level of WT, and *C9orf72*^***−/−***^ mice had no detectable *C9orf72* expression (Supplemental Fig. 1B). Finally, we confirmed no difference in transcription levels of nearby loci *Mob3b*, *Ak045932*, and *Ifnk*, indicating insertion of *lacZ* impacts *C9orf72* expression only (Supplemental Fig. 1C, data not shown).

Consistent with Taqman results, staining for *lacZ* in tissues from 6 and 28 week *C9orf72*^***−/−***^ revealed enzyme activity in the brain, spinal cord, spleen, testes, and kidney, corresponding to previously published findings^23^,^25^. We also observed staining in additional tissues, including fat, muscle, atria, liver, and lung (Supplemental Fig. 1D, data not shown). Reporter activity was more limited in intensity and scope in *C9orf72*^+/−^ tissues, as expected for a single lacZ replacement allele.

### *C9orf72* ^*−/−*^mice show mild motor deficits

Given the association of C9ORF72 to neuropathology, we performed clinical exams^26^ to determine whether loss of *C9orf72* causes an ALS-like phenotype. At 40 weeks of age, *C9orf72*^***−/−***^showed progressive weakness and collapse of hind limbs towards the lateral midline, with mild tremor and rigidity not observed in WT or *C9orf72*^+/−^ (Supplemental Fig. 2A, data not shown). Open field observations demonstrated decreased locomotor behaviors and fewer rearing events in null mice compared with WT. CatWalk gait analyses also revealed signs of impaired lower interlimb coordination and reduced stride length with bradykinesia and dragging of hind limbs (Supplemental Fig. 2B). No difference between WT and *C9orf72*^***−/−***^ mice was observed in maximum time spent on the rotarod (Supplemental Fig. 2C).

### *C9orf72* ^*−/−*^ mice exhibit lymphadenopathy and splenomegaly

**Figure 1:**
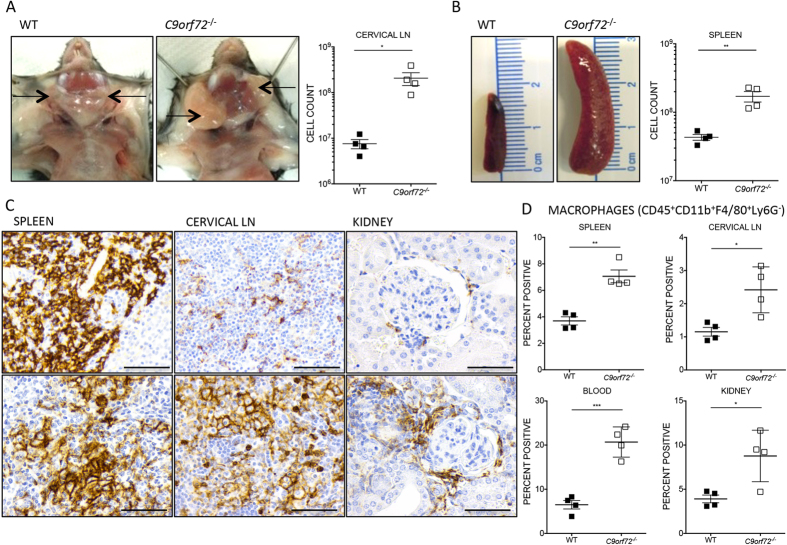
*C9orf72*^***−/−***^ mice develop lymphadenopathy and splenomegaly, and display infiltration of F4/80^+^ cells by IHC and FACS Analysis. (**A**,**B**) Representative pictures of gross cervical LN enlargement and splenomegaly observed in *C9orf72*^***−/−***^in comparison to age-matched WT control. Significantly increased cell counts obtained via FACS analysis correspond to lymphadenopathy and splenomegaly observed grossly. **(C)** The expanded cell populations infiltrating the red pulp of the spleen and surrounding lymphoid follicles of the cervical LN stained positive by IHC for mouse macrophage marker F4/80. Periglomerular infiltrates observed in *C9orf72*^***−/−***^ kidneys are also largely positive for F4/80 macrophage lineage marker. Sections shown are females, 37 week old *C9orf72*^***−/−***^ and 40 week old WT **(D)** FACS analysis confirmed H&E and IHC findings by showing increased percentages of CD11b^+^F4/80^+^Ly6G^−^ macrophages in kidney, spleen, cervical LN, and blood (30–35 week old female, n = 4 per genotype). **(A**–**D)** Data are shown as mean ± s.e.m (*P ≤ 0.05, **P ≤ 0.01 and ***P ≤ 0.001 by unpaired Students *t*-test). **(C)** Scale bar represents 50 μm, original magnification, ×600.

During neurological testing, we unexpectedly noted palpable cervical masses in all *C9orf72*^***−/−***^ animals, but not in WT or *C9orf72*^+/−^ controls. Masses were palpable as early as 8 weeks of age and present in all null mice by 18 weeks, before onset of observed motor deficits. Upon dissection, the masses proved to originate from cervical lymph node (LN) (Fig. 1A, data not shown) and systemic lymphadenopathy was noted in certain null mice. Peyer’s patches (PP) were also enlarged and splenomegaly was apparent by 8 weeks of age (Fig. 1B, data not shown). By 36 weeks, *C9orf72*^***−/−***^ ceased gaining weight compared with WT and only 9 out of 17 survived to the end of the neurological assay period (60 weeks) (Supplemental Fig. 2D, data not shown).

### *C9orf72* ^*−/−*^ mice display mixed inflammatory infiltrates in multiple organs

The enlargement of *C9orf72*^***−/−***^ spleens and LN suggests a disease process such as neoplasm or immune dysregulation, an unexpected finding given that ALS/FTD is not linked to such pathology in human patients. To address these possibilities, histopathology was conducted on spleen and LN from 8–60 week old mice. The basic cellular organization of the enlarged *C9orf72*^***−/−***^ LN was preserved, with immunohistochemistry (IHC) identifying a B cell-rich rim (CD45R^+^) arranged in follicles within the cortex and a T cell (CD3^+^) infiltrate between follicles and in the paracortex zone (data not shown). A mixed cell population consisting mostly of large round cells with variably distinct borders, a single round nucleus, and eosinophilic, foamy cytoplasm expanded the cortical and medullary nodal architecture. A similar cellular infiltrate was present in *C9orf72*^***−/−***^ spleen, predominantly located within the red pulp, expanding the splenic architecture and corresponding to grossly increased spleen weights. These cells did not stain consistently with CD45R or CD3 but were strongly positive for the macrophage lineage marker F4/80. (Fig. 1C, data not shown). Abundant plasmacytoid cells containing perinuclear halos, consistent with plasma cell morphology, and occasional Mott cells (enlarged plasma cells containing cytoplasmic immunoglobulin (Ig) inclusions) were also present. We did not observe similar infiltrates in WT and *C9orf72*^+/−^ controls.

H&E and IHC analyses of additional organs from mice aged 8–60 weeks revealed a prominent F4/80^+^ population of elongated to angular cells in the liver and kidneys of null mice. This population was pronounced in *C9orf72*^***−/−***^ mouse liver at 8 weeks, though there was no evidence of associated liver disease (data not shown). Increased F4/80^+^ cell populations observed in *C9orf72*^***−/−***^ kidney were located primarily within the cortex, forming prominent cuffs around glomeruli and aggregates in the vicinity of the macula densa and adjacent tubules (Fig. 1C). Increasing infiltrates of mixed leukocytes were also observed with age, accompanied by varying degrees of immune-mediated glomerular disease that was well established by 35–60 weeks. Inflammation was not present in brain or spinal cord tissue in any animals examined. These data implicate the spleen, LN and kidney as major sites of *C9orf72*^***−/−***^ immune pathology.

### *C9orf72* ^*−/−*^ lymphoid organs contain increased percentages of myeloid lineage cells

To further interrogate the cellular infiltrate observed on histopathology, flow cytometric analysis was performed on spleen, cervical and mesenteric LN, PP, BM, blood and kidney from *C9orf72*^***−/−***^ and WT controls. Specific focus was on the 28–35 week time point in females, as the majority show renal pathology but remain viable. Total CD45^+^ (leukocyte common antigen) cell counts were increased in all *C9orf72*^***−/−***^ tissues examined, consistent with the immune infiltration described above, however CD45^+^ percentages compared with total cell populations assayed were either unchanged or reduced compared with WT. To determine if homeostasis within leukocyte subsets was altered, we narrowed our focus using specific antibody panels. F4/80^+^ macrophages (CD45^+^CD11b^+^F4/80^+^Ly6G^−^) were increased in the spleen, LN, kidney, and blood in *C9orf72*^***−/−***^ mice, consistent with the F4/80^+^ infiltration observed by IHC (Fig 1D). Neutrophil (CD45^+^CD11b^+^Ly6G^+^Ly6C^int^CD115^−^) and total monocyte (CD45^+^CD11b^+^CD115^+^) percentages were also increased in *C9orf72*^***−/−***^ tissues compared with controls. Staining for Ly6G and Ly6C revealed an increased percentage of inflammatory monocytes (CD45^+^CD11b^+^CD115^+^Ly6G^−^Ly6C^hi^) in *C9orf72*^***−/−***^ spleen, LN, blood, and kidney. Myeloid dendritic cell (CD45^+^CD11b^+^CD11c^+^MHCII^+^) percentages measured in the spleen, cervical LN, mesenteric LN, PP, BM, and blood were increased in *C9orf72*^***−/−***^ relative to WT, whereas the NK cell (NKp46^+^CD49b^+^) fraction was decreased in spleen and BM. Co-staining for the activation marker CD86 revealed activated myeloid DC populations (CD45^+^CD11b^+^CD11c^+^MHCII^+^CD86^+^) in all tissues examined (data not shown). Similar perturbations in myeloid cell populations by FACS analyses on males aged 9–60 weeks are summarized in Supplemental Fig. 3 with representative FACS plots provided in Supplemental Fig. 4. Complete Blood Count (CBC) fractionation of leukocyte populations also revealed increases in circulating neutrophils, monocytes, and eosinophils in *C9orf72*^***−/−***^ compared with controls, in addition to significant anemia and thrombocytopenia (data not shown).

### IL-12, IL-17a, IL-10, and TNF are increased in *C9orf72* ^*−/−*^ serum

**Figure 2:**
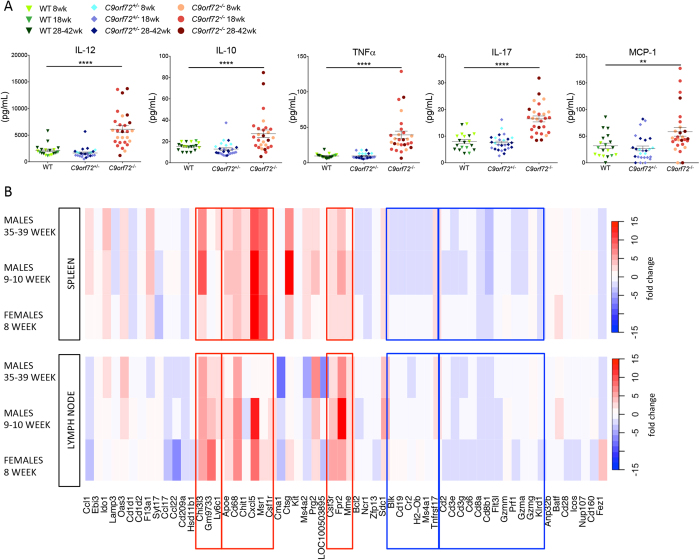
Serum cytokine data and molecular profiling signatures indicate myeloid cell infiltration and involvement of macrophage activating pathways in *C9orf72*^*−/−*^ mice. (**A**) *C9orf72*^***−/−***^ show increased levels of circulating cytokines and chemokines involved with macrophage recruitment and activation. *C9orf72*^+/−^ mice demonstrate values comparable to WT, consistent with absence of any observed phenotype. Graphs represent mean ± s.e.m. (**P ≤ 0.01 and ****P ≤ 0.0001 by one-way ANOVA) from 8–38 week female mice, n ≥ 19 per genotype. (**B**) Molecular profiling signatures from *C9orf72*^***−/−***^ spleen and cervical LN suggest infiltration of macrophage, monocyte, and granulocyte cell populations by increased expression of associated markers. Depletion of T and B cells is also indicated, which may reflect the increase in proportion of myeloid cells. Data shown is from 8 week old females, 9–10 week old males, and 35–39 week old males, n ≥ 3 per genotype.

To further characterize the global effects of C9orf72 ablation, we measured various cytokines and chemokines in 8–60 week mouse serum (Fig. 2A, data not shown). IL-12total was approximately 6-fold increased in *C9orf72*^***−/−***^ animals compared with controls. IL-10, IL-17a, and TNFα were also upregulated, although to a lesser extent. We did not observe changes in IL-1β, IL-2, or IL-4 suggesting this effect on cytokine secretion was not global. While there was a trend toward increased IL-6 in *C9orf72*^***−/−***^ serum compared with WT, this difference did not reach statistical significance. MCP-1 chemokine was significantly increased in female *C9orf72*^***−/−***^ animals and IFNγ was significantly increased in males with an increasing trend observed in females.

### RNAseq analyses reveal global inflammatory gene signatures in *C9orf72* ^*−/−*^ mice

*C9orf72* ablation appears to cause a systemic immune response resulting in elevated inflammatory cytokines and myeloid cell expansion. High expression of *C9orf72* has been observed in monocytes, macrophages and DCs, with lower levels measured in lymphocytes. C9orf72 may indeed modulate the immune system, particularly the myeloid compartment (Supplemental Fig. 5)^27^. To confirm this observation, we performed RNASeq analyses, mapping global transcriptome changes between WT and *C9orf72*^***−/−***^ brain and lymphoid tissues. In the brain, hierarchical clustering primarily separated samples by gender and age, indicating that profiling differences in this tissue are due to the basic biology of samples (data not shown). In contrast, spleen and LN samples clustered based on genotype, with age and sex secondary, thus transcriptome differences in these organs result from loss of *C9orf72* expression. Furthermore, over 100 loci associated with immune function showed significant expression differences between *C9orf72*^***−/−***^ and WT tissues in both genders and ages. *C9orf72*^***−/−***^ spleen and LN gene signatures indicate myeloid infiltration with a decrease in the lymphocytic signature. (Fig. 2B). A NextBio enrichment analysis of this sample set revealed the strongest perturbations in gene sets involved in immune response, mouse models of inflammatory conditions, and human infectious diseases, consistent with *C9orf72* ablation resulting in global immune dysregulation.

### *C9orf72* ^*−/−*^ mice have increased percentages of activated T lymphocytes and plasma cells

**Figure 3:**
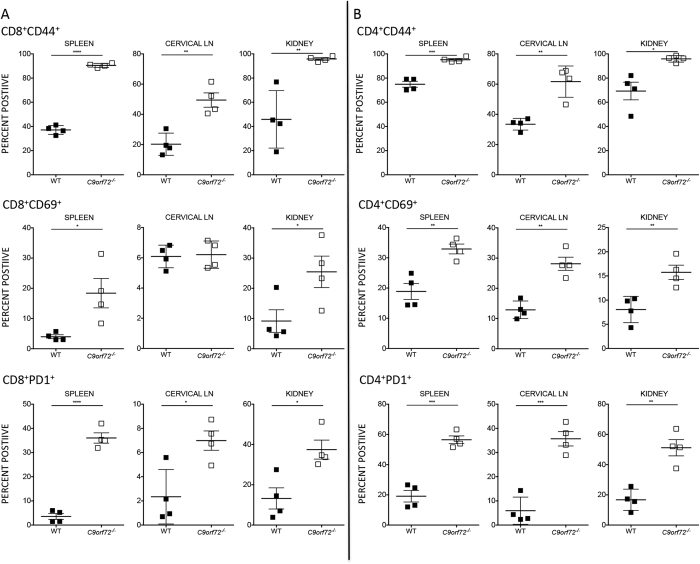
*C9orf72*^*−/−*^ mice show increased expression of T cell activation markers by FACS analysis. (**A**) CD8^+^ T cell summary data reflect increases in the percentage of CD8 T cells expressing the activation markers CD44 and CD69 and the co-inhibitory receptor PD-1 in *C9orf72*^***−/−***^ compared with WT. (**B**) Similarly, CD4^+^ T cell summary data represent increased percentages of *C9orf72*^***−/−***^ CD4 T cells expressing CD44, CD69, and PD1 in spleen, cervical LN, and kidney with varying significance. Graphs represent mean ± s.e.m. (*P ≤ 0.05, **P ≤ 0.01, ***P ≤ 0.001, ****P ≤ 0.0001 by unpaired Students *t*-test) 30–35 week females, n = 4 per genotype.

An increase in activation markers on monocytic cells is often accompanied by increases in lymphocyte activation parameters. In addition, the elevation in IL-12 observed in *C9orf72*^***−/−***^ serum could up regulate T cell activity^28^. To quantify T cell populations, we performed flow cytometric analyses on spleens, LN, BM, blood and kidney from 30–35 week *C9orf72*^***−/−***^ and WT female mice, gating on CD45 leukocyte common antigen, CD4 or CD8, and various activation status markers. Percentages of CD45^+^CD8^+^ and CD45^+^CD4^+^ cells were reduced overall in *C9orf72*^***−/−***^ animals compared with controls, likely a consequence of myeloid expansion and consistent with RNA profiling results. Conversely, but reflective of expansion of lymphoid tissue and immune infiltration, total cell counts for these T cell populations were increased in null mice (data not shown). Co-staining CD8^+^ T cells with activation markers revealed increases in the early activation and effector memory T cell markers CD69 and CD44 in *C9orf72*^***−/−***^ spleen and kidney compared with controls. We observed significantly increased percentages of CD8^+^ T cells expressing PD-1, a co-inhibitory receptor up regulated on activated cells with an important role in down-regulating the immune system. Cervical LN showed significantly increased expression of CD44 and PD-1, although CD69 expression was relatively unchanged (Fig. 3A). CD44, CD69, and PD-1 expression was also increased on CD4^+^ T cells in *C9orf72*^***−/−***^ spleen, LN, and kidney (Fig. 3B). We also measured less pronounced increases in activated T cell populations in the blood and BM (data not shown). CD4^+^ FoxP3^+^ regulatory T (Treg) cell percentages were elevated in spleens and LN from *C9orf72*^***−/−***^ animals compared with WT (data not shown). Finally, reduced expression of the naïve and central memory markers CD62L and CD127 in *C9orf72*^***−/−***^ spleens corroborates ongoing immune activation, as these molecules are down-regulated once cells become activated (Supplemental Fig. 6A). Total cell counts for activated T cell populations are depicted in Supplemental Fig. 6B,C and representative FACS plots are shown in Supplemental Fig. 7A.

**Figure 4:**
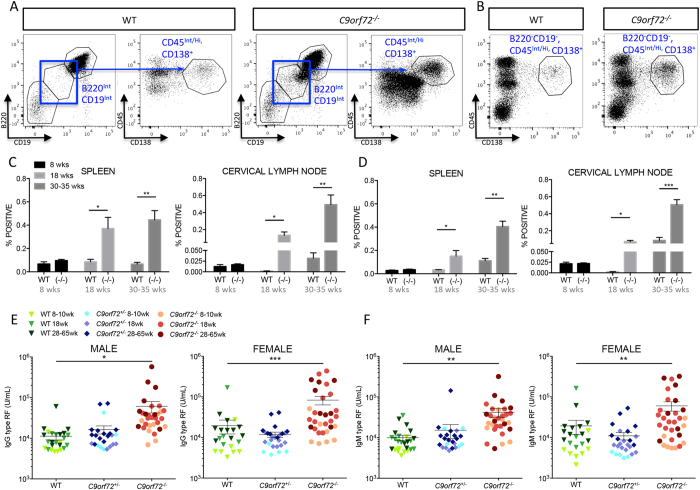
Increased percentages of plasma cells and autoantibody production contribute to autoimmune dysfunction in *C9orf72*^*−/−*^ mice. (**A**) FACS analysis reveals a population of cells transitioning from mature B cells to plasma cells indicated by a decrease in expression of mature B cell markers, CD19 and B220 (blue box), and concomitant strong expression of the mature plasma cell marker CD138. Gating strategy from WT and *C9orf72*^***−/−***^ 18 week male spleen is represented to show a transitioning plasma cell population that is prominent in *C9orf72*^***−/−***^ compared with WT. (**B**) Gating strategy for a mature plasma cell population (B220^−^CD19^−^CD45^Int/Hi^CD138^+^) in 18 week male spleen demonstrates increased mature plasma cells in *C9orf72*^***−/−***^compared with WT. (**C**) Graphical representation of increased transitioning and (**D**) mature plasma cell populations by FACS analysis shows significantly increased percentages in *C9orf72*^***−/−***^spleen and LN in comparison to WT at 18 weeks of age and older. (**E**,**F**) Serum ELISA assays indicate significant increases in IgG and IgM type RF autoantibodies, consistent with the observed systemic autoimmune response in null mice. *C9orf72*^+/−^ mice display values comparable to WT, consistent with absence of any observed phenotype. (**C**,**D**) Graphs represent mean ± s.e.m. (*P ≤ 0.05, **P ≤ 0.01 and ***P ≤ 0.001 by unpaired Students *t*-test), n = 4 females per genotype (**E**,**F**) Graphs represent mean ± s.e.m. (*P ≤ 0.05, **P ≤ 0.01 and ***P ≤ 0.001 by one-way ANOVA), n ≥ 22 per genotype, 9–65 week old males and 8–42 week old females.

B cell (CD45^+^B220^+^CD19^+^) percentages were either unchanged or reduced in *C9orf72*^***−/−***^ spleen but increased in LN compared with controls. Similar to T cell populations, total B cell counts were increased overall in spleen and LN, reflective of lymphoid expansion (data not shown). Analysis of specific B cell subsets revealed significantly increased marginal zone (MZ), (CD45^+^CD19^+^B220^+^CD21^+^CD23^−^), follicular (FO) (CD45^+^CD19^+^B220^+^CD21^+/−^CD23^+^CD93^−^), and germinal center (GC) (CD45^+^CD19^+^B220^+^CD38^−^IgD^−^GL7^+^Fas^+^) B cells in *C9orf72*^***−/−***^ cervical LN compared with WT. B cell populations in null spleens were overall unchanged or reduced however, similar to myeloid DC, staining for the activation marker CD86 demonstrated an increased activated B cell population (CD45^+^CD19^+^B220^+^CD86^+^) in both *C9orf72*^***−/−***^ spleen and LN (Supplemental Fig. 8A,B). Representative FACS plots are provided in Supplementary Fig. 8C. CD138 co-staining demonstrated increased percentages of mature plasma cells (CD45^+^CD19^−^B220^−^CD138^+^) in *C9orf72*^***−/−***^ LN, spleen, and BM, and an expanded population of B cells transitioning to plasma cells (CD45^+^CD19^int^B220^int^CD138^+^) in all three tissues (Fig. 4A–D and data not shown). We did not find consistent differences between *C9orf72*^***−/−***^ and WT in these cell types in the blood (data not shown). Taken together, FACS analyses reveal increases in myeloid and lymphoid cell populations, and upregulation in *C9orf72*^***−/−***^ T and B cell activation markers indicative of a systemic immune response.

### *C9orf72* ^*−/−*^ mice have high titers of autoantibodies

Expansions in plasma cells and transitioning B cells/plasmablasts can be associated with neoplasms such as multiple myeloma and plasmacytoma^29^,^30^, in addition to autoimmunity^31^,^32^. The spleen and LN of *C9orf72*^***−/−***^ animals were enlarged by infiltrates of F4/80^+^ foamy macrophages occupying appropriate regions for their lineage without obliteration of tissue architecture. Despite showing abnormal proliferation, the mitotic index was low, with only rare mitoses (data not shown). Thus, while a pre-neoplastic condition cannot be excluded, the presence of plasma cells, occasional Mott cells, and glomerulonephritis are more indicative of a potential primary autoimmune process. Serum chemistry panels demonstrated elevated globulin in *C9orf72*^***−/−***^ mice compared with controls and ELISAs showed significantly increased total IgG and IgM in *C9orf72*^***−/−***^ mouse serum (data not shown). We therefore tested WT, *C9orf72*^+/−^, and *C9orf72*^***−/−***^ serums for the common autoantibody, anti-rheumatoid factor (RF), high titers of which are linked to a variety of autoimmune disorders^33^. Both IgG and IgM-type anti-RF titers were significantly elevated in *C9orf72*^***−/−***^ serum compared with controls (Fig. 4E,F).

### Aging *C9orf72* ^*−/−*^ mice exhibit varying degrees of proliferative glomerulonephropathy

**Figure 5:**
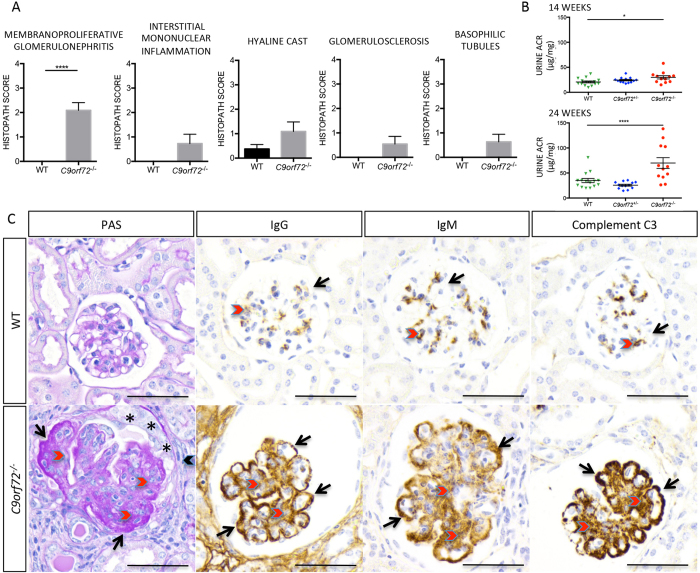
*C9orf72*^***−/−***^ mice show evidence of progressive glomerulonephropathy (**A**) Weighted graphs of histopathological scoring demonstrate the most significant renal changes observed in *C9orf72*^***−/−***^ mice are associated with membranoproliferative glomerulonephritis. **(B)** Urine ACR measurements assayed at 14 and 24 week timepoints from the same cohort of mice indicate onset of albuminuria in *C9orf72*^***−/−***^ animals with age. Heterozygous mice display values comparable to WT consistent with the absence of an observed phenotype. (**C**) PAS Staining and IHC immunoreactivity of mouse glomeruli for IgG, IgM, and C3 demonstrate immune-mediated kidney damage. Mesangial matrix (red chevron) is PAS+ and markedly expanded in *C9orf72*^***−/−***^ mouse compared with the delicate matrix seen in the WT. Basement membrane of the vasculature is markedly expanded by PAS+ matrix (black arrows), obliterating the capillary loop. The parietal epithelium is proliferative (*), and Bowman’s capsule is surrounded by connective tissue and mononuclear cells (black chevron). IHC for IgG, IgM, and C3 in WT (top panel) shows delicate capillary loops (black arrows) supported by scant mesangial matrix; faint chromogenic staining of the capillary endothelia is representative of physiologic IgG, IgM, and C3. IHC stippling is also observed in the mesangium (red chevrons). In contrast, glomeruli from *C9orf72*^***−/−***^ mice have increased mesangial matrix (red chevrons) and granular deposits of IgG, IgM, and C3. Capillary loop basement membranes are thickened and delineated by subendothelial/subepithelial granular to confluent deposits (black arrows). Chromogenic staining for IgG is also apparent in surrounding renal tissue. Note the increased size of the *C9orf72*^***−/−***^ glomerulus compared with that of the WT, and the increase in the urinary space of the glomerulus. **(A**,**C)** Data represented is from 35–63 week old females, n ≥ 8 per genotype analyzed. Scale bar represents 50 μm, original magnification, ×600. **(A)** Graphs represent mean ± s.e.m. (***P ≤ 0.0001 by non-parametric Mann-Whitney) **(B)** Graphs represent mean ± s.e.m. (*P ≤ 0.05, ***P ≤ 0.0001 by one way ANOVA).

As prefaced earlier, F4/80^+^ monocytes were present in high numbers in *C9orf72*^***−/−***^ kidneys (Fig. 1C) with evidence of progressive glomerular disease observed by histopathology. To further characterize renal changes in null mice, H&E stained kidney sections were analyzed and scored in five categories of disease. Results showed significantly higher average scoring for membranoproliferative glomerulonephritis in *C9orf72*^***−/−***^ with evidence of occasional glomerulosclerosis, hyaline casts, basophilic tubules and interstitial mononuclear inflammation compared with WT controls (Fig. 5A). Individual histopath scores are shown in Supplemental Table 1. H&E staining of the renal cortex (Supplemental Fig. 9) is representative of mild (middle row) to marked (bottom row) disease progression. Descriptively, mild changes consisted of glomerular enlargement with increased cellularity, and enlargement of Bowman’s space. Moderate to severely affected animals also had tubular changes, increased interstitial leukocytic infiltration, thickened capillary walls, and proliferation of visceral (podocytes) and parietal epithelium. In 3/11 null mice, glomerulosclerosis was present, characterized by expansion of the mesangial matrix with acellular, eosinophilic hyaline material, and a variable degree of periglomerular fibrosis. Tubular changes observed in 4/11 *C9orf72*^***−/−***^ kidneys included basophilic tubules (with degeneration/regeneration), cortical and medullary tubular dilatation, hyaline proteinaceous casts, and interstitial infiltrates of mononuclear cells. Consistent with impaired glomerular filtration and correlative to histopathological renal findings, a serum chemistry panel revealed significant elevation of blood urea nitrogen (BUN) and decreased serum albumin in *C9orf72*^***−/−***^ compared with controls (data not shown). Onset of albuminuria is also indicated by urinary albumin to creatinine ratios (ACR) assayed at 14 and 24 week time points. Elevated ACR observed at 24 weeks in null mice is indicative of a progressing renal disease course (Fig. 5B).

Deposition of soluble immune complexes within the glomerular capillaries, followed by complement fixation can cause renal disease such as immune-mediated glomerulonephropathy^34^ (GN). High magnification periodic acid schiff (PAS) staining represented in Fig. 5C demonstrated regional thickening of the glomerular basement membrane in capillary loops associated with immune-mediated GN in *C9orf72*^***−/−***^ mice, but not WT. To further evaluate whether increased total Ig and autoantibody levels contribute to GN, we performed IHC on *C9orf72*^***−/−***^ and WT, 8–63 week kidney sections for total IgG and IgM as well as complement C3 on a 60–63 week surviving cohort of females. Overall, *C9orf72*^***−/−***^ kidneys demonstrated increased IgG immunostaining compared with WT at all time points, with diffuse, intense IHC signal in the vasculature and tubular epithelium of the medulla and cortex observed as early as 8 weeks. Correlating with the onset of GN pathology, we noted an increase in glomerular IgG and IgM staining by 38 weeks (Fig. 5C, Supplemental Fig. 9). Staining for both IgG and IgM was also frequently associated with the parietal layer of Bowman’s capsule. IgG was occasionally observed in the urinary space and/or within proximal renal tubules, indicating impaired glomerular filtration. Intense IgG staining was present in tubule epithelial cells in animals with severe disease, consistent with reabsorption of abundant IgG. Occasionally, similar, less intense staining was observed for IgM. Staining in sclerotic glomeruli was diminished compared with WT, consistent with impaired blood flow to these units. However, glomeruli that retained patent vascular loops tended to have increased IgG in comparison with WT. Fine granular deposits and/or linear staining of IgG and IgM associated with vascular membranes suggestive of immune complex deposition were frequently present (Fig. 5C). Complement factor C3 deposition is commonly associated with immunoglobulin deposits on basement membranes in immune-mediated glomerular disease^34^. IHC for C3 revealed increased staining in glomerular tufts of *C9orf72*^***−/−***^ mice compared with WT (Supplemental Fig. 9A). Granular and linear staining was most prominent on the membranes of the visceral layer of glomerular capsule, delineating the capillary loops and podocytes as depicted in 60× magnification (Fig. 5C).

### *C9orf72* ^*−/−*^ mice develop SLE-like disease

**Figure 6:**
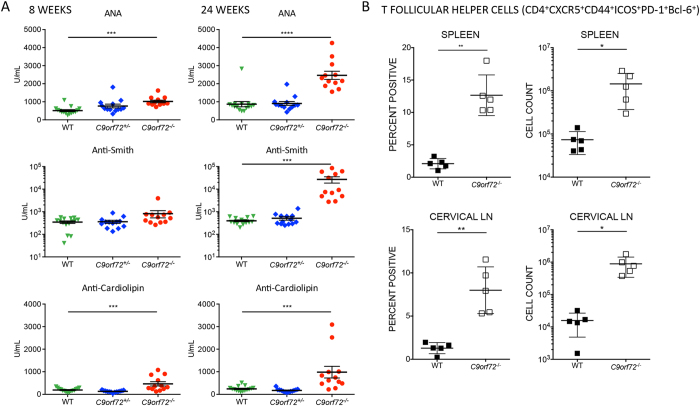
*C9orf72*^*−/−*^ mice show increased serum autoantibodies and increased T follicular helper cells reminiscent of human SLE. **(A)** Serum ELISAs from the same cohort of mice assayed at 8 and 24 weeks of age demonstrate elevated levels of circulation ANA and SLE-specific autoantibodies, anti-Sm, and anti-cardiolipin as early as 8 weeks in *C9orf72*^***−/−***^mice. At 24 week timepoint, all three autoantibodies are significantly increased in null mice with fold increases observed in comparison to WT. Heterozygous mice display values comparable to WT consistent with the absence of an observed phenotype. **(B)** Tfh cells (CD4^+^CXCR5^+^CD44^+^ICOS^+^PD-1^+^Bcl-6^+^) are significantly increased by percent and total cell count in *C9orf72*^***−/−***^ spleen and cervical LN **(A)** Graphs represent mean ± s.e.m. (***P ≤ 0.001 and ****P ≤ 0.0001 by one-way ANOVA), 8 and 24 week females, n ≥ 12 per genotype. **(B)** Graphs represent mean ± s.e.m. (*P ≤ 0.05, **P ≤ 0.01 by unpaired Students *t*-test), 26 week females, n = 5 per genotype.

Systemic lupus erythematosis (SLE) is characterized by immune dysregulation affecting many organs of the body^35^. *C9orf72*^***−/−***^ mice develop the lymphoid hyperplasia, anemia, and renal disease common in SLE patients and reminiscent of phenotypes observed in spontaneous mouse models of SLE such as the MRL/lpr and NZB/W F1 strains^36^. A hallmark of SLE is high titer antinuclear antibodies (ANA) of certain specific types^37^. We therefore tested *C9orf72*^***−/−***^ and control mouse serum for ANA encompassing a subset of autoantibodies against proteins and structures in the nucleus; specifically, we tested for anti-Smith (Sm) antibodies that recognize core units of small nuclear ribonucleic proteins (snRNP), anti-double-stranded DNA (dsDNA) antibodies, as well as anti-cardiolipin antibodies that are reactive to an essential element of the inner mitochondrial membrane. ANA, anti-dsDNA and anti-cardiolipin were significantly increased in female *C9orf72*^***−/−***^ mice by 8 weeks compared with controls. Anti-Sm was slightly elevated at 8 weeks in null mice with a dramatic increase above WT observed by the 24 week timepoint (Fig. 6A, data not shown).

Increased autoantibody titers in lupus patients are positively correlated with an increased frequency of circulating T follicular helper (Tfh) cells^38^. Interrogation of this specific cell population (CD4^+^CXCR5^+^CD44^+^ICOS^+^PD-1^+^Bcl-6^+^) in spleen, cervical LN, mesenteric LN, and blood by FACS analysis revealed significantly increased Tfh cell populations in *C9orf72*^***−/−***^ tissues compared with controls. (Fig. 6B data not shown). Elevated Tfh cells were also observed in *C9orf72*^***−/−***^ BM that did not reach significance (data not shown). Collectively, our data suggest an immune response similar to human SLE occurs in the absence of *C9orf72* expression.

## Discussion

The association between *C9ORF72* GGGGCC repeat expansion and neurological disease is well-established, but the pathogenic mechanism remains elusive. The absence of phenotype in *C9orf72*^+/−^ mice presented in our study contradicts haploinsufficiency of C9ORF72 as the main cause of C9ALS/FTD pathology. More importantly, our study highlights a novel immune regulatory role for C9orf72 through the first comprehensive phenotypic analysis of a mouse line with global C9orf72 ablation. Since analog immunological findings are not typically present among ALS patients, our data indicate that *C9orf72* gene function is unrelated to known C9ALS/FTD pathology, making a nonspecific effect of the repeat expansion on C9ALS/FTD pathology more likely.

Global ablation of C9orf72 resulted in select expansions of myeloid and lymphoid compartments, with increased T, B and DC cell activation and elevated plasma cells. *C9orf72*^***−/−***^ mice demonstrated elevated serum IL-12 and other cytokines, in addition to tissue RNA signatures consistent with myeloid upregulation. Renal disease with accompanying pathological changes was present in the majority of mice by 35 weeks. At a microscopic level, glomeruli stained heavily with antibody to Ig and C3 in a pattern suggesting immune complex deposition. Null mice also had increased Tfh cell populations and high titer anti-RF, ANA, anti-Sm, and anti-cardiolipin autoantibodies that are commonly associated with human SLE^37^,^38^. In summary, loss of *C9orf72* expression profoundly disturbs immune homeostasis and results in systemic autoimmune disease.

SLE is a constellation of immunological abnormalities affecting multiple organ systems. Patients may have manifestations in the kidney, skin, joints, lungs, and/or heart that usually follow a fluctuating course, with periodic flares and periods of reduced disease activity^35^. 95–98% of patients have elevated ANA and an elevated anti-Sm antibody titer is pathognomonic for SLE^39^. Mouse models of SLE include the spontaneously occurring MRL/lpr and NZB/W F1 strains, as well as other inducible models, however none fully represent human disease^36^. NZB/W F1 mice replicate the female bias toward worsened pathology, elevated ANA with anti-dsDNA and immune complex-mediated glomerulonephritis, but do not develop anti-Sm ribonuclear protein antibodies. MRL/lpr mice have inflammation in many classic SLE sites and elevated anti-Sm and anti-cardiolipin, although males and females are equally affected. Neither of these strains or any single-gene or inducible model replicates the waxing and waning in symptoms characteristic of human disease. *C9orf72*^***−/−***^ animals display a subset of lupus-like symptoms, namely lymphoid activation and hyperplasia, a characteristic autoantibody profile and evidence of GN. They do not replicate all aspects of human SLE and the same caveats relevant to established models should also be applied to our mice.

Human SLE may also comprise neuropsychiatric symptoms, including cognitive dysfunction, mood disorders, psychosis, and cerebrovascular diseases, collectively referred to as neuropsychiatric SLE (NP-SLE). Onset of symptoms may be associated with high titer autoantibodies, increased blood-brain barrier (BBB) permeability, and cytokine production^40^. The MRL/lpr mouse strain develops behavioral abnormalities and is commonly used as a model to study such manifestations^41^. High titer anti-cardiolipin correlates positively with neuropsychiatric symptoms^42^,^43^ and is observed in both MRL/lpr and our *C9orf72*^***−/−***^ mice. We observed mild motor impairment in *C9orf72*^***−/−***^ that suggested neurological deficit, however lymphoid organ hyperplasia and GN were already established, suggesting defects could be secondary to the immune phenotype. We did not observe overt pathology or marked transcriptome changes in *C9orf72*^***−/−***^ brain and spinal cord, however we cannot rule out highly localized disease processes not obvious by our assays. Given the growing body of evidence supporting a link between autoimmune mechanisms and neurological disease^44^, studies to evaluate behavioral and cognitive disorders in *C9orf72*^***−/−***^ mice should also be considered.

*C9orf72* expression profiling in immune cell types assembled by the Immunological Genome Project (Immgen) and additional microarray expression studies reflect high expression of *C9orf72* in monocyte, macrophages and DC populations^27^,^45^. Relative to this insight, expanded proliferative potential of the monocyte system with increased F4/80^+^ macrophages has previously been described in MRL/lpr and NZB/W mice. These strains show early involvement of the BM in monocytopoiesis, and retain up-regulated extramedullary monocyte proliferation, in contrast to control strains that down-regulate this process as the BM develops its full activity^46^. Defects in phagocytosis intrinsic to F4/80^+^ cells have been described in mouse SLE models and human patients^47^,^48^ and inefficient clearance of apoptotic debris is considered a hallmark of SLE^49^. Indeed, polymorphisms in the autophagy-related gene, *ATG5* are linked to increased susceptibility to SLE^50^,^51^, as is activation of the negative regulator of autophagy, mTOR^52^. Alternatively, other studies have reported enhanced autophagy in patient and mouse model B cells^53^,^54^ and patient serum factors are capable of inducing autophagy in neuroblastoma cell lines^55^. We have not yet assessed the phagocytic potential of the predominating F4/80^+^ cells in *C9orf72*^***−/−***^ mice, but such experiments could determine whether dysregulation of autophagy is associated with C9orf72 ablation and contributing to the SLE phenotype or is simply a byproduct of other processes.

C9ORF72 protein is widely expressed and highly conserved as a single-gene copy across all vertebrates with remarkably high sequence identity across species. Notably, it has not been duplicated, even in fish genome, all of which have undergone rounds of duplication suggesting its function is crucial, but under constraint. Although still largely unknown, C9ORF72 function has been linked to intracellular trafficking via Rab-dependent pathways essential for endosomal transport. Depletion of C9ORF72 by siRNA in neuronal cell lines inhibited endocytosis and dysregulated autophagy, an important process for cellular homeostasis^56^. Given that autophagy can protect against neurodegenerative disease by preventing accumulation of toxic proteins, disrupting this process by reducing C9ORF72 expression may render cells more susceptible to repetitive RNA and the products of erroneous hexanucleotide repeat translation.

Recent studies using human C9ORF72 isoform-specific antibodies demonstrated colocalization of the short isoform with components of the nuclear pore complex. Interestingly, motor neurons derived from C9ALS/FTD patients exhibited loss of the short C9ORF72 isoform and mislocalization of TDP-43, indicating defects in nucleocytoplasmic shuttling^57^. More recently, however, three studies suggest the repeat expansion itself can affect shuttling independent of its surrounding genetic locus. Yeast expressing dipeptide inclusions and *Drosophilia* expressing RNA repeats presented with defects in intracellular transport, a finding then replicated in repeat-transfected cell lines, patient inducible pluripotent stem cell neurons and C9ALS–FTD patient brain tissue^58^,^59^,^60^. To date, all relevant protein function and repeat-associated studies have been restricted to neuronal cell lines and tissue samples. Further experimentation in a wider array of cell types could help to elucidate a specific role for C9ORF72 and separate processes relevant to protein function from those associated with the repeat. Furthermore, pursuing similar experiments outside the CNS could reveal novel relationships between autoimmunity and neurodegeneration and unveil new pathways with therapeutic potential.

In summary, our results implicate loss of mouse C9orf72 expression with autoimmunity that resembles human SLE and suggest a new role for C9ORF72 as an important regulator of the immune system. *C9ORF72* has not yet been linked to SLE in humans and pursuit of potential disease variants with a specific focus on autoimmune populations should be considered. Further experimentation in cell lines outside the CNS should also be undertaken to help elucidate the function of C9ORF72. In addition, we propose loss of C9orf72 protein is not the likely cause of C9ALS/FTD neuropathology. Generation of a mouse model with the repeat expansion targeted into the *C9orf72* locus to more closely mimic the human genetic lesion will help to address C9ALS/FTD pathological mechanisms.

## Methods

### Generation of *C9orf72* ^*−/−*^ mice

We employed the VelociGene® and VelociMouse® methods as described previously^61^,^62^,^63^,^64^ in which targeted ES cells (F1 hybrid 129S6SvEvTac/C57BL6NTac ES cells) were injected into uncompacted 8-cell stage Swiss Webster embryos to produce healthy fully ES cell-derived F0 generation mice heterozygous (Het) for the *C9orf72* mutation. F0 generation male Hets were crossed with C57Bl6/NTac females to generate F1 Hets that were intercrossed to produce F2 generation WT, *C9orf72*^+/−^ and *C9orf72*^***−/−***^ mice for phenotypic analyses. A second cohort of N2F2 generation mice was generated via *in-vitro* fertilization (IVF) using frozen F1 heterozygous sperm and oocytes from C57Bl6/NTac donor females. N2F1 Het offspring were then intercrossed to generate N2F2 WT, *C9orf72*^+/−^ and *C9orf72*^***−/−***^ mice for phenotypic analysis.

### Animals

Phenotypic studies of F2 and N2F2 mice began at 6 weeks of age. Mice were observed from birth for various developmental milestones (runting, breathing, facial and limb abnormalities, skin color, posture, righting and eye opening) until 6 weeks of age, when they were housed 2–5 per cage in 12 hours of light per day at 20–23 °C, and 40–60% humidity for study. Mice were housed in 95.6 × 309.1 × 133.4 mm cages (Thoren) with cob bedding (The Andersons Lab Bedding) and a cotton nestlet for enrichment (Ancare). Mice had access to normal chow (LabDiet) and water ad libitum and were monitored twice daily for health status. All animal procedures were carried out in strict accordance with the recommendations in the Guide for the Care and Use of Laboratory Animals of the National Institutes of Health and were approved by the Regeneron Pharmaceuticals Institutional Animal Care and Use Committee (IACUC).

### Taqman expression analysis

Axillary and brachial lymph nodes, cervical lymph nodes, gonadal fat pad, frontal cortex, diaphragm, spinal cord, spleen, and thymus were dissected fresh into RNALater stabilization reagent (Qiagen) and stored at −20 °C. Tissues were homogenized in TRIzol and chloroform was used for phase separation. The aqueous phase, containing total RNA, was purified using miRNeasy Mini Kit (Qiagen, Cat#217004) according to manufacturer’s specifications. Genomic DNA was removed using MagMAX™Turbo™DNase Buffer and TURBO DNase (Ambion by Life Technologies). mRNA was reverse-transcribed into cDNA using SuperScript® VILO™ Master Mix (Invitrogen by Life Technologies, Cat# 11755500). cDNA was amplified with the TaqMan® Gene Expression Master Mix (Applied Biosystems by Life Technologies, Cat# 4370074) using the ABI 7900HT Sequence Detection System (Applied Biosystems). *Actb* was used as the internal control gene to normalize cDNA input differences. WT thymus was used as a reference sample to calculate the fold difference of mRNA between samples.

### LacZ expression profiling

Mice were deeply anesthetized via Ketamine/Xylazine (120/5 mg/kg) intraperitoneal (IP) injection and fixed by cardiac perfusion using a 0.2% glutaraldehyde, 4% paraformaldehyde (PFA) solution. Brain, ribcage, lymph nodes, salivary glands, thymus, heart, lung, liver, spleen, stomach, kidney, intestine, urogenital, muscle, and hind limb tissues were dissected, rinsed in phosphate buffered saline (PBS) and post-fixed for 30 minutes in a 0.2% glutaraldehyde, 4% PFA solution. Tissues were washed and incubated in X-gal (1 mg/mL) staining solution for roughly 12 hours at 37 °C. After staining, tissues were washed, post-fixed in 4% PFA and cleared in a series of 50%, 70% and 100% glycerol. Photographs were taken with a Nikon SMZ1500 stereomicroscope and Nikon DS-Ri1 digital camera using NIS-Elements D Imaging Software (Nikon).

### Behavioral scoring tests

The assessment of overall motor function was performed using blinded subjective scoring assays. Motor impairment score was measured using the system generated by ALS Therapy Development Institute (ALS TDI)^26^. Tremor and rigidity scores were measured using a 0–3 scale, where 0 = no symptoms, 1 = mild, 2 = moderate, and 3 = severe. Locomotor behaviors were evaluated for 60 minutes every other week using the automated Open Field system (Kinder Scientific), which measures fine movements, X+Y ambulation, distance traveled, number of rearing events, time spent rearing, and immobility time via infrared beam breaks. Rotarod (IITC Life Science, Woodland Hills, CA) was used to measure the latency for a mouse to fall from a rotating beam with a ramping speed, starting at 1 rpm and accelerating to 15 rpm over 180 seconds. The average and maximum of the three longest durations of time that the animals stay on the beam without falling were used to evaluate latency. Gait analysis was performed using the CatWalk XT 10 (Noldus). Mice spontaneously ambulated across a runway and the footprints were automatically analyzed for interlimb coordination (the percentage of normal step sequences) and stride length (the distance between successive placements of the same paw).

### Cell preparation and FACS analysis

Blood was collected into heparin-coated tubes by cardiac puncture immediately following CO_2_ euthanization. Spleen, BM, cervical LN, mesenteric LN, and PP were harvested and dissociated into single cell suspensions in Dulbecco’s 1× PBS with 2% fetal bovine serum, (Stem Cell Technologies), plus 2 mM EDTA, (Ambion). Red blood cell (RBC) lysis was performed using 1× RBC lysis buffer (eBioscience) or ACK Lysing Buffer (Life Technologies). All staining was performed using LIVE/DEAD Fixable Aqua or Blue Stain (Invitrogen; 15 minutes at room temperature) and Fc block (Purified Rat Anti-Mouse CD16/CD32; BD Pharmingen, 5 minutes at room temperature). Surface staining was completed with the indicated directly conjugated antibodies for 30 minutes on ice. Foxp3 and Bcl-6 staining (eBioscience) were executed according to the manufacturers instructions. Antibodies to CD3, CD4, CD8, CD11b, CD11c, CD25, CD45, B220, CD62L, CD44, CD69, CD127, PD1 (RPMI-30), NKp46, Ly6C, and Ly6G were purchased from BioLegend (San Diego, CA). Antibodies to CD115, Bcl-6 and Foxp3 were purchased from eBioscience (San Diego, CA). Antibodies to CD19, CD49b, CD138, F4/80, CXCR5, ICOS, were purchased from BD Biosciences (San Jose CA). Samples were fixed with 1× Stabilizing fixative and collected using FACSCanto or Fortessa Flow Cytometers (BD Biosciences, San Jose, CA). Data were analyzed by FlowJo Software (Tree Star).

### Histology

Tissues were directly harvested into 4% PFA or collected following transcardial perfusion with 50 mL of saline solution, 50 mL of 4% PFA solution in acetate buffer at pH 6.5 and finally 50 ml of 4% PFA solution in borate buffer at pH 9.5. Spinal cords were collected into 15% followed by 30% sucrose solution in borate buffer until they sank. All other tissues were post-fixed in 4% PFA and transferred to 70% ethanol after 24 hours. Paraffin embedding, sectioning, and H&E staining were performed by Histoserv, Inc. (Germantown, MD). PAS staining and IHC for IgG, IgM, C3, CD45R, CD3, CD138, and F4/80 was completed by Histotox Labs, (Boulder, CO). Histopathological scoring of H&E stained kidney sections was performed by a blinded, board certified veterinary pathologist and evaluated in disease categories according to guidelines set forth by The International Harmonization of Nomenclature and Diagnostic Criteria for Lesions in Rats and Mice (INHAND) Project^65^.

### Hematology assays

Blood samples were collected by either retro-orbital eye bleeds under isoflurane anesthesia or cardiac puncture after euthanasia by CO_2_ inhalation in accordance with Regeneron IACUC protocol. CBC with differential was performed on 20 μL of whole blood using Hemavet 950 (Drew Scientific Group) and clinical chemistry was completed on serum samples using ADVIA 1800 Chemistry System (Siemans Medical Solutions USA). ELISAs were performed on plasma samples using the following; Mouse IgG and IgM Rheumatoid Factor ELISA Kit (Shibayagi Co., Ltd), Mouse Anti-dsDNA Total Ig ELISA kit, Mouse ANA Total Ig ELISA kit, Mouse Anti-Sm Total Ig ELISA kit, Mouse Anti-Cardiolipin Total Ig ELISA kit (Alpha Diagnostic Intl.), and IgG and IgM mouse ELISA kit (Abcam) as per the manufacturer’s instructions. IFN-γ, IL-1β, IL-2, IL-4, IL-6, IL-10, IL-12total, IL-17, MCP-1, and TNF-α were measured in plasma samples using a Multi-Spot® 10plex electrochemiluminescence detection assay (Meso Scale Discovery) according to the manufacturer’s instructions.

### Urinalysis

Urine samples were obtained via spot collection and urinary albumin concentration was determined with Albuwell M indirect competitive ELISA kit (Exocell, Philadelphia, PA). Urinary creatinine concentration was assayed using the Creatinine Companion kit (Exocell). Assays were performed according to manufacturer’s instructions and data obtained were used to calculate the urine albumin-to-creatinine ratio.

### RNA isolation, sequencing and analysis

Spleen and cervical lymph nodes were dissected fresh into RNALater stabilization reagent (Qiagen) and stored at −20 °C. Total RNA was isolated using MagMAX™ Nucleic Acid Isolation Kit (Ambion) per the manufacturer’s instructions. RNA was quantified using UV spectrophotometer, and RNA integrity was evaluated by Qiaxcel (Qiagen). PolyA mRNA was purified from total RNA using Dynabeads mRNA kit (Invitrogen) and strand specific RNA-Seq libraries were prepared with the ScriptSeq RNA-seq Library Preparation kit (Illumina). RNA-Seq libraries were sequenced to a length of 33 bp using Hiseq 2000 NGS sequencer (Illumina). Gene expression levels were derived from raw sequencing reads using Nimbus2, an RNA-Seq analysis pipeline developed in house.

### Statistical analysis

Statistical and graphical analyses were performed using GraphPad Prism software (version 3.0). Data were analyzed using unpaired Student’s *t*-test, one-way analysis of variance (ANOVA) and non-parametric Mann-Whitney as indicated. Results were considered statistically significant at P values < 0.05.

## Additional Information

**How to cite this article**: Atanasio, A. *et al.* *C9orf72* ablation causes immune dysregulation characterized by leukocyte expansion, autoantibody production, and glomerulonephropathy in mice. *Sci. Rep.* **6**, 23204; doi: 10.1038/srep23204 (2016).

## Supplementary Material

Supplementary Information

## References

[b1] DeJesus-Hernandez M. *et al.* Expanded GGGGCC hexanucleotide repeat in noncoding region of C9ORF72 causes chromosome 9p-linked FTD and ALS. Neuron 72, 245–256 (2011). 21944778 10.1016/j.neuron.2011.09.011 PMC3202986

[b2] Renton A. E. *et al.* A hexanucleotide repeat expansion in C9ORF72 is the cause of chromosome 9p21-linked ALS-FTD. Neuron 72, 257–268 (2011). 21944779 10.1016/j.neuron.2011.09.010 PMC3200438

[b3] van Swieten J. C. & Grossman M. FTD/ALS families are no longer orphaned: the C9ORF72 story. Neurology 79, 962–964 (2012). 22875094 10.1212/WNL.0b013e318268471a

[b4] Donnelly C. J., Grima J. C. & Sattler R. Aberrant RNA homeostasis in amyotrophic lateral sclerosis: potential for new therapeutic targets? Neurodegener. Dis. Manag. 4, 417–437 (2014). 25531686 10.2217/nmt.14.36 PMC4308292

[b5] Haeusler A. R. *et al.* C9orf72 nucleotide repeat structures initiate molecular cascades of disease. Nature 507, 195–200 (2014). 24598541 10.1038/nature13124 PMC4046618

[b6] Lee Y. B. *et al.* Hexanucleotide repeats in ALS/FTD form length-dependent RNA foci, sequester RNA binding proteins, and are neurotoxic. Cell Rep 5, 1178–1186 (2013). 24290757 10.1016/j.celrep.2013.10.049 PMC3898469

[b7] Mori K. *et al.* hnRNP A3 binds to GGGGCC repeats and is a constituent of p62-positive/TDP43-negative inclusions in the hippocampus of patients with C9orf72 mutations. Acta Neuropathol 125, 413–423 (2013). 23381195 10.1007/s00401-013-1088-7

[b8] Porta S., Kwong L. K., Trojanowski J. Q. & Lee V. M. Drosha inclusions are new components of dipeptide-repeat protein aggregates in FTLD-TDP and ALS C9orf72 expansion cases. J Neuropathol Exp Neurol 74, 380–387 (2015). 25756586 10.1097/NEN.0000000000000182 PMC4362478

[b9] Sareen D. *et al.* Targeting RNA foci in iPSC-derived motor neurons from ALS patients with a C9ORF72 repeat expansion. Sci Transl Med 5, 208ra149 (2013). 10.1126/scitranslmed.3007529 PMC4090945 24154603

[b10] Xu Z. *et al.* Expanded GGGGCC repeat RNA associated with amyotrophic lateral sclerosis and frontotemporal dementia causes neurodegeneration. Proc Natl Acad Sci USA 110, 7778–7783 (2013). 23553836 10.1073/pnas.1219643110 PMC3651485

[b11] Ash P. E. *et al.* Unconventional translation of C9ORF72 GGGGCC expansion generates insoluble polypeptides specific to c9FTD/ALS. Neuron 77, 639–646 (2013). 23415312 10.1016/j.neuron.2013.02.004 PMC3593233

[b12] Mori K. *et al.* Bidirectional transcripts of the expanded C9orf72 hexanucleotide repeat are translated into aggregating dipeptide repeat proteins. Acta Neuropathol 126, 881–893 (2013). 24132570 10.1007/s00401-013-1189-3

[b13] Mori K. *et al.* The C9orf72 GGGGCC repeat is translated into aggregating dipeptide-repeat proteins in FTLD/ALS. Science 339, 1335–1338 (2013). 23393093 10.1126/science.1232927

[b14] Zu T. *et al.* RAN proteins and RNA foci from antisense transcripts in C9ORF72 ALS and frontotemporal dementia. Proc Natl Acad Sci USA 110, E4968–4977 (2013). 24248382 10.1073/pnas.1315438110 PMC3870665

[b15] Stancu I. C., Vasconcelos B., Terwel D. & Dewachter I. Models of beta-amyloid induced Tau-pathology: the long and “folded” road to understand the mechanism. Mol Neurodegener 9, 51 (2014). 25407337 10.1186/1750-1326-9-51 PMC4255655

[b16] Gijselinck I. *et al.* A C9orf72 promoter repeat expansion in a Flanders-Belgian cohort with disorders of the frontotemporal lobar degeneration-amyotrophic lateral sclerosis spectrum: a gene identification study. Lancet Neurol 11, 54–65 (2012). 22154785 10.1016/S1474-4422(11)70261-7

[b17] Waite A. J. *et al.* Reduced C9orf72 protein levels in frontal cortex of amyotrophic lateral sclerosis and frontotemporal degeneration brain with the C9ORF72 hexanucleotide repeat expansion. Neurobiol Aging 35, 1779 e1775-1779 e1713 (2014). 10.1016/j.neurobiolaging.2014.01.016 PMC3988882 24559645

[b18] Fratta P. *et al.* C9orf72 hexanucleotide repeat associated with amyotrophic lateral sclerosis and frontotemporal dementia forms RNA G-quadruplexes. Sci Rep 2, 1016 (2012). 23264878 10.1038/srep01016 PMC3527825

[b19] Reddy K., Zamiri B., Stanley S. Y., Macgregor R. B. Jr. & Pearson C. E. The disease-associated r(GGGGCC)n repeat from the C9orf72 gene forms tract length-dependent uni- and multimolecular RNA G-quadruplex structures. J Biol Chem 288, 9860–9866 (2013). 23423380 10.1074/jbc.C113.452532 PMC3617286

[b20] Sket P. *et al.* Characterization of DNA G-quadruplex species forming from C9ORF72 G4C2-expanded repeats associated with amyotrophic lateral sclerosis and frontotemporal lobar degeneration. Neurobiol Aging 36, 1091–1096 (2015). 25442110 10.1016/j.neurobiolaging.2014.09.012

[b21] Ciura S. *et al.* Loss of function of C9orf72 causes motor deficits in a zebrafish model of amyotrophic lateral sclerosis. Ann Neurol 74, 180–187 (2013). 23720273 10.1002/ana.23946

[b22] Therrien M., Rouleau G. A., Dion P. A. & Parker J. A. Deletion of C9ORF72 results in motor neuron degeneration and stress sensitivity in C. elegans. PLoS One 8, e83450 (2013). 24349511 10.1371/journal.pone.0083450 PMC3861484

[b23] Koppers M. *et al.* C9orf72 ablation in mice does not cause motor neuron degeneration or motor deficits. Ann Neurol 78, 426–438 (2015). 26044557 10.1002/ana.24453 PMC4744979

[b24] Lagier-Tourenne C. *et al.* Targeted degradation of sense and antisense C9orf72 RNA foci as therapy for ALS and frontotemporal degeneration. Proc Natl Acad Sci USA 110, E4530–4539 (2013). 24170860 10.1073/pnas.1318835110 PMC3839752

[b25] Suzuki N. *et al.* The mouse C9ORF72 ortholog is enriched in neurons known to degenerate in ALS and FTD. Nat Neurosci 16, 1725–1727 (2013). 24185425 10.1038/nn.3566 PMC4397902

[b26] Gill A., Kidd J., Vieira F., Thompson K. & Perrin S. No benefit from chronic lithium dosing in a sibling-matched, gender balanced, investigator-blinded trial using a standard mouse model of familial ALS. PLoS One 4, e6489 (2009). 19649300 10.1371/journal.pone.0006489 PMC2714460

[b27] Heng T. S., Painter M. W. & Immunological Genome Project, C. The Immunological Genome Project: networks of gene expression in immune cells. Nat Immunol 9, 1091–1094 (2008). 18800157 10.1038/ni1008-1091

[b28] Vignali D. A. & Kuchroo V. K. IL-12 family cytokines: immunological playmakers. Nat Immunol 13, 722–728 (2012). 22814351 10.1038/ni.2366 PMC4158817

[b29] Kilciksiz S., Karakoyun-Celik O., Agaoglu F. Y. & Haydaroglu A. A review for solitary plasmacytoma of bone and extramedullary plasmacytoma. Scientific World Journal 2012, 895765 (2012). 22654647 10.1100/2012/895765 PMC3354668

[b30] Palumbo A. & Anderson K. Multiple myeloma. N Engl J Med 364, 1046–1060 (2011). 21410373 10.1056/NEJMra1011442

[b31] Tarlinton D. M. & Hodgkin P. D. Targeting plasma cells in autoimmune diseases. J Exp Med 199, 1451–1454 (2004). 15173204 10.1084/jem.20040719 PMC2211780

[b32] Browning J. L. B cells move to centre stage: novel opportunities for autoimmune disease treatment. Nat Rev Drug Discov 5, 564–576 (2006). 16816838 10.1038/nrd2085

[b33] Ingegnoli F., Castelli R. & Gualtierotti R. Rheumatoid factors: clinical applications. Dis Markers 35, 727–734 (2013). 24324289 10.1155/2013/726598 PMC3845430

[b34] Kurts C., Panzer U., Anders H. J. & Rees A. J. The immune system and kidney disease: basic concepts and clinical implications. Nat Rev Immunol 13, 738–753 (2013). 24037418 10.1038/nri3523

[b35] Apostolidis S. A., Lieberman L. A., Kis-Toth K., Crispin J. C. & Tsokos G. C. The dysregulation of cytokine networks in systemic lupus erythematosus. J Interferon Cytokine Res 31, 769–779 (2011). 21877904 10.1089/jir.2011.0029 PMC3189553

[b36] Perry D., Sang A., Yin Y., Zheng Y. Y. & Morel L. Murine models of systemic lupus erythematosus. J Biomed Biotechnol 2011, 271694 (2011). 21403825 10.1155/2011/271694 PMC3042628

[b37] Sherer Y., Gorstein A., Fritzler M. J. & Shoenfeld Y. Autoantibody explosion in systemic lupus erythematosus: more than 100 different antibodies found in SLE patients. Semin Arthritis Rheum 34, 501–537 (2004). 15505768 10.1016/j.semarthrit.2004.07.002

[b38] Xu H. *et al.* Increased frequency of circulating follicular helper T cells in lupus patients is associated with autoantibody production in a CD40L-dependent manner. Cell Immunol 295, 46–51 (2015). 25748125 10.1016/j.cellimm.2015.01.014

[b39] Migliorini P., Baldini C., Rocchi V. & Bombardieri S. Anti-Sm and anti-RNP antibodies. Autoimmunity 38, 47–54 (2005). 15804705 10.1080/08916930400022715

[b40] Kivity S., Agmon-Levin N., Zandman-Goddard G., Chapman J. & Shoenfeld Y. Neuropsychiatric lupus: a mosaic of clinical presentations. BMC Med 13, 43 (2015). 25858312 10.1186/s12916-015-0269-8 PMC4349748

[b41] Gulinello M. & Putterman C. The MRL/lpr mouse strain as a model for neuropsychiatric systemic lupus erythematosus. J Biomed Biotechnol 2011, 207504 (2011). 21331367 10.1155/2011/207504 PMC3038428

[b42] Zandman-Goddard G., Chapman J. & Shoenfeld Y. Autoantibodies involved in neuropsychiatric SLE and antiphospholipid syndrome. Semin Arthritis Rheum 36, 297–315 (2007). 17258299 10.1016/j.semarthrit.2006.11.003

[b43] Sciascia S., Bertolaccini M. L., Roccatello D., Khamashta M. A. & Sanna G. Autoantibodies involved in neuropsychiatric manifestations associated with systemic lupus erythematosus: a systematic review. J Neurol 261, 1706–1714 (2014). 24952022 10.1007/s00415-014-7406-8

[b44] Zivkovic S. Autoimmune neurologic disorders. Curr Neuropharmacol 9, 399 (2011). 22379453 10.2174/157015911796557993 PMC3151593

[b45] Nataf S. & Pays L. Gene co-expression analysis unravels a link between C9orf72 and RNA metabolism in myeloid cells. Acta Neuropathol Commun 3, 64 (2015). 26472214 10.1186/s40478-015-0242-y PMC4608290

[b46] Muller M., Emmendorffer A. & Lohmann-Matthes M. L. Expansion and high proliferative potential of the macrophage system throughout life time of lupus-prone NZB/W and MRL lpr/lpr mice. Lack of down-regulation of extramedullar macrophage proliferation in the postnatal period. Eur J Immunol 21, 2211–2217 (1991). 1889463 10.1002/eji.1830210932

[b47] Herrmann M. *et al.* Impaired phagocytosis of apoptotic cell material by monocyte-derived macrophages from patients with systemic lupus erythematosus. Arthritis Rheum 41, 1241–1250 (1998). 9663482 10.1002/1529-0131(199807)41:7<1241::AID-ART15>3.0.CO;2-H

[b48] Potter P. K., Cortes-Hernandez J., Quartier P., Botto M. & Walport M. J. Lupus-prone mice have an abnormal response to thioglycolate and an impaired clearance of apoptotic cells. J Immunol 170, 3223–3232 (2003). 12626581 10.4049/jimmunol.170.6.3223

[b49] Shao W. H. & Cohen P. L. Disturbances of apoptotic cell clearance in systemic lupus erythematosus. Arthritis Res Ther 13, 202 (2011). 21371352 10.1186/ar3206 PMC3157636

[b50] Zhou X. J. *et al.* Genetic association of PRDM1-ATG5 intergenic region and autophagy with systemic lupus erythematosus in a Chinese population. Ann Rheum Dis 70, 1330–1337 (2011). 21622776 10.1136/ard.2010.140111

[b51] Zhang Y. M. *et al.* Rare Variants of ATG5 Are Likely to Be Associated With Chinese Patients With Systemic Lupus Erythematosus. Medicine (Baltimore) 94, e939 (2015). 26039132 10.1097/MD.0000000000000939 PMC4616363

[b52] Fernandez D. R. *et al.* Activation of mammalian target of rapamycin controls the loss of TCRzeta in lupus T cells through HRES-1/Rab4-regulated lysosomal degradation. J Immunol 182, 2063–2073 (2009). 19201859 10.4049/jimmunol.0803600 PMC2676112

[b53] Clarke A. J. *et al.* Autophagy is activated in systemic lupus erythematosus and required for plasmablast development. Ann Rheum Dis 74, 912–920 (2015). 24419333 10.1136/annrheumdis-2013-204343 PMC4152192

[b54] Pierdominici M. *et al.* Role of autophagy in immunity and autoimmunity, with a special focus on systemic lupus erythematosus. FASEB J 26, 1400–1412 (2012). 22247332 10.1096/fj.11-194175

[b55] Towns R. *et al.* Sera from patients with type 2 diabetes and neuropathy induce autophagy and colocalization with mitochondria in SY5Y cells. Autophagy 1, 163–170 (2005). 16874076 10.4161/auto.1.3.2068

[b56] Farg M. A. *et al.* C9ORF72, implicated in amytrophic lateral sclerosis and frontotemporal dementia, regulates endosomal trafficking. Hum Mol Genet 23, 3579–3595 (2014). 24549040 10.1093/hmg/ddu068 PMC4049310

[b57] Xiao S. *et al.* Isoform-specific antibodies reveal distinct subcellular localizations of C9orf72 in amyotrophic lateral sclerosis. Ann Neurol 78, 568–583 (2015). 26174152 10.1002/ana.24469

[b58] Freibaum B. D. *et al.* GGGGCC repeat expansion in C9orf72 compromises nucleocytoplasmic transport. Nature 525, 129–133 (2015). 26308899 10.1038/nature14974 PMC4631399

[b59] Jovicic A. *et al.* Modifiers of C9orf72 dipeptide repeat toxicity connect nucleocytoplasmic transport defects to FTD/ALS. Nat Neurosci 18, 1226–1229 (2015). 26308983 10.1038/nn.4085 PMC4552077

[b60] Zhang K. *et al.* The C9orf72 repeat expansion disrupts nucleocytoplasmic transport. Nature 525, 56–61 (2015). 26308891 10.1038/nature14973 PMC4800742

[b61] Dechiara T. M. *et al.* VelociMouse: fully ES cell-derived F0-generation mice obtained from the injection of ES cells into eight-cell-stage embryos. Methods Mol Biol 530, 311–324 (2009). 19266341 10.1007/978-1-59745-471-1_16

[b62] DeChiara T. M. *et al.* Producing fully ES cell-derived mice from eight-cell stage embryo injections. Methods Enzymol 476, 285–294 (2010). 20691872 10.1016/S0076-6879(10)76016-X

[b63] Poueymirou W. T. *et al.* F0 generation mice fully derived from gene-targeted embryonic stem cells allowing immediate phenotypic analyses. Nat Biotechnol 25, 91–99 (2007). 17187059 10.1038/nbt1263

[b64] Valenzuela D. M. *et al.* High-throughput engineering of the mouse genome coupled with high-resolution expression analysis. Nat Biotechnol 21, 652–659 (2003). 12730667 10.1038/nbt822

[b65] Frazier K. S. *et al.* Proliferative and nonproliferative lesions of the rat and mouse urinary system. Toxicol Pathol 40, 14S–86S (2012). 22637735 10.1177/0192623312438736

