# Supplementary material for: C9orf72 ablation causes immune dysregulation characterized by leukocyte expansion, autoantibody production, and glomerulonephropathy in mice
Source: Sci Rep. 2016 Mar 16;6:23204. doi: 10.1038/srep23204 (PMC4793236; doi:10.1038/srep23204)
Supplement: Supplementary Information [file srep23204-s1.pdf]

# *C9orf72* ablation causes immune dysregulation characterized by leukocyte expansion, autoantibody production, and glomerulonephropathy in mice

**Amanda Atanasio[1], Vilma Decman[1], Derek White[1], Meg Ramos[1], Burcin Ikiz[1], Hoi-Ching Lee[1], Chia-Jen Siao[1], Susannah Brydges[1], Elizabeth LaRosa[1], Yu Bai[1], Wen Fury[1], Patricia Burfeind[1], Ralica Zamfirova[1], Gregg Warshaw[1], Jamie Orengo[1], Adelekan Oyejide[1], Michael Fralish[1], Wojtek Auerbach[1], William Poueymirou[1], Jan Freudenberg[1], Guochun Gong[1], Brian Zambrowicz[1], David Valenzuela[1], George Yancopoulos[1], Andrew Murphy[1], Gavin Thurston[1], & Ka-Man Venus Lai[1*]**

[1] Regeneron Pharmaceuticals, Inc., 777 Old Saw Mill River Road, Tarrytown, NY 10591

* Correspondence should be addressed to: K-MVL [Venus.Lai@regeneron.com]

# Supplemental Figure 1

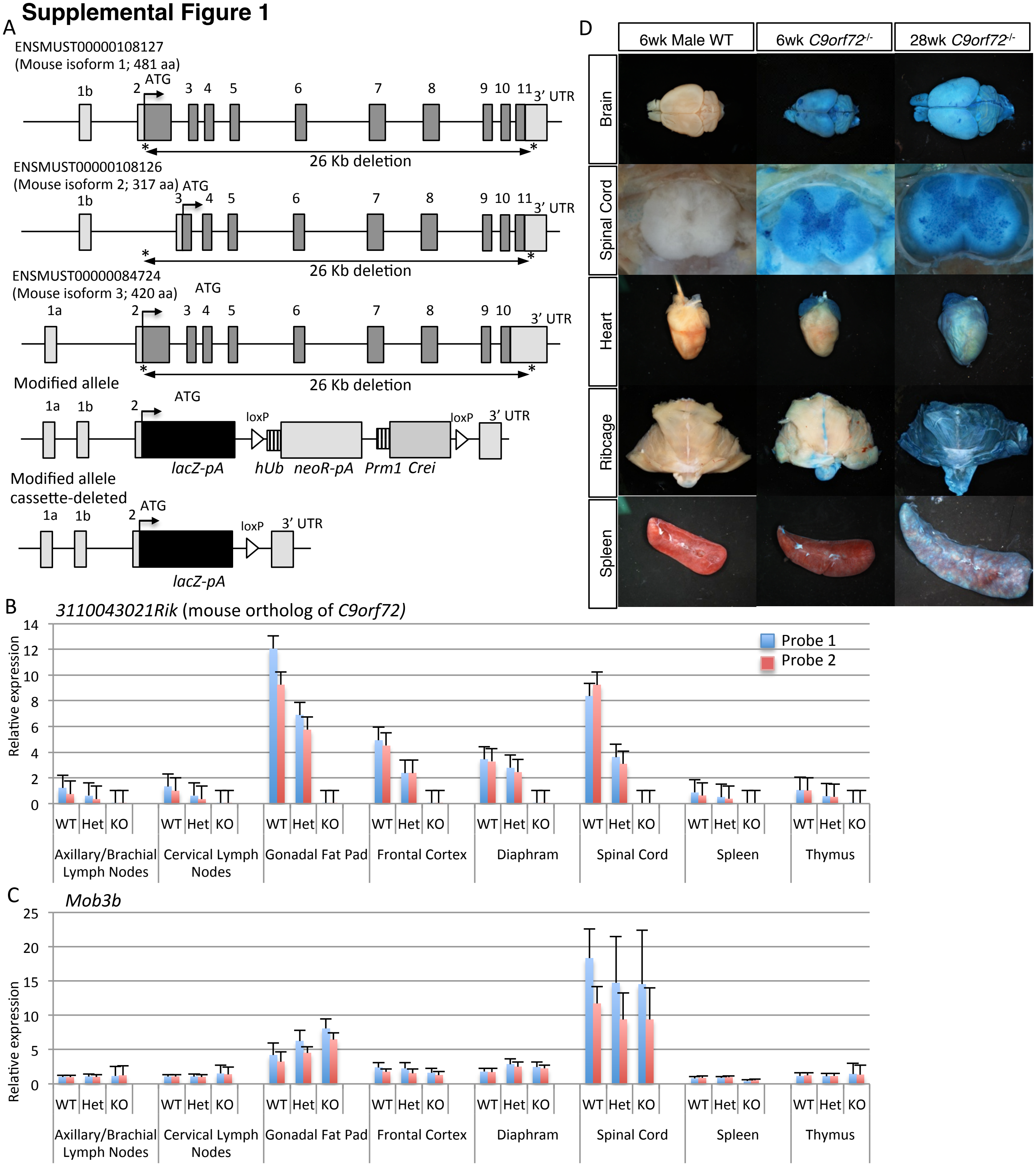


**Supplemental Fig. 1** *C9orf72* targeting strategy and Taqman expression analysis. (**A**) *C9orf72* coding sequence and intervening introns were replaced with a *lacZ* floxed neomycin resistance cassette. Self-deleting technology was employed to remove the neomycin cassette prior to phenotypic analysis, leaving the *lacZ* expression reporter and one loxP site under control of the *C9orf72* promoter (manuscript in preparation). (**B**) Taqman expression analysis of *C9orf72* confirms ablation of C9orf72 and reveals WT and *C9orf72*$^{+/-}$ mRNA expression levels in CNS, muscle, fat and lymphoid organs. (**C**) Taqman expression analysis confirms mRNA levels of neighboring gene *Mob3b* are unaffected by targeted deletion of *C9orf72*. (**D**) *LacZ* expression profiling reveals widespread staining in the brain and spinal cord as well as atria, spleen, and muscle in 6 and 28 week *C9orf72*$^{-/-}$ mice. (**B,C**) Results were normalized to expression of the control gene *Actb* and are presented relative to WT thymus expression. Data are shown as mean and standard deviation (s.d.) of n=5 females per genotype for each tissue.

# Supplemental Figure 2

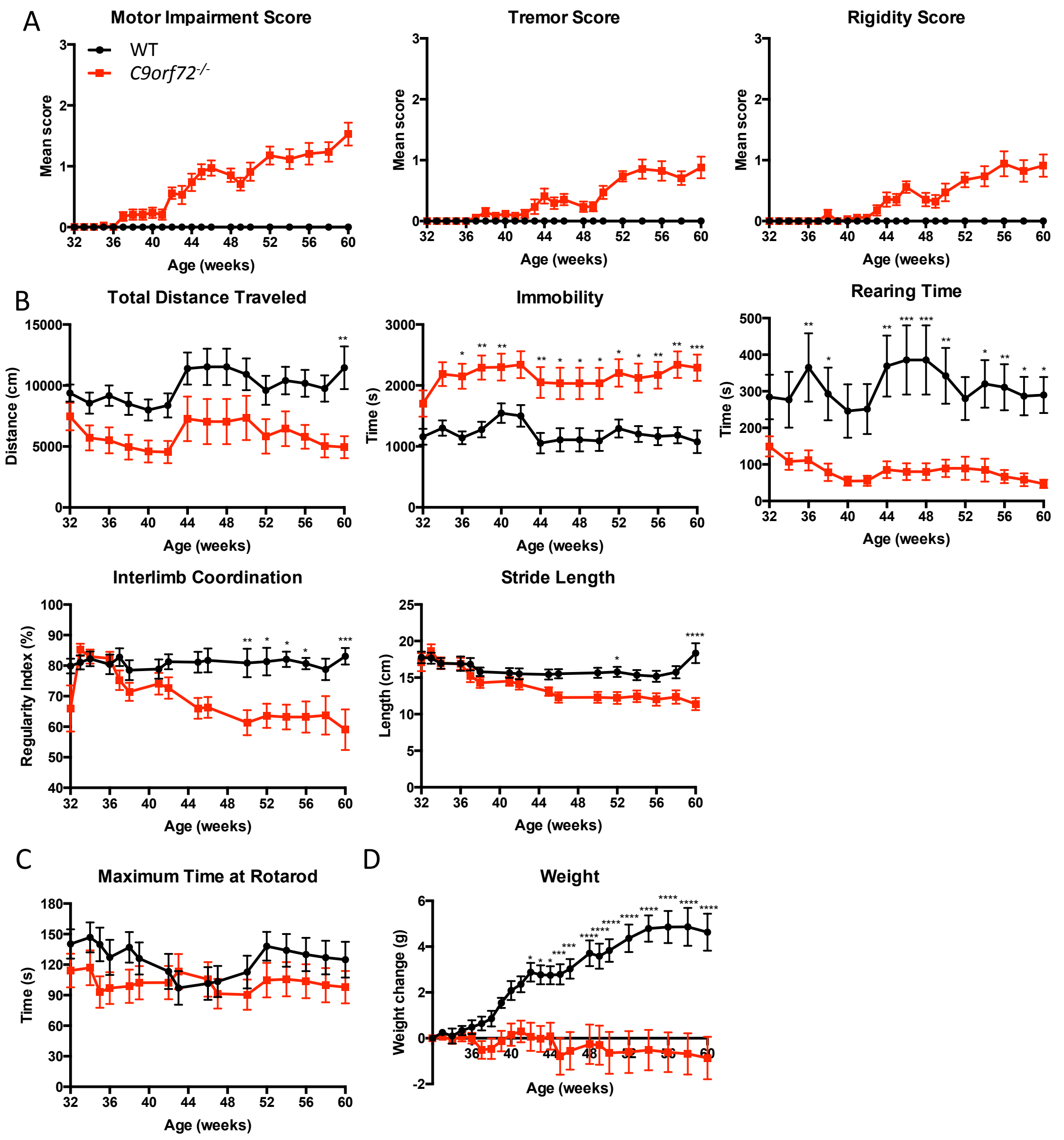


**Supplemental Fig. 2** Aging *C9orf72*$^{-/-}$ mice show signs of motor impairment with decreased locomotor behavior and gait abnormalities. (**A**) Beginning at 40 weeks of age, *C9orf72*$^{-/-}$ mice begin to show mild indications of motor impairment with increased tremor and rigidity of hind limbs compared with WT. (**B**) Automated open field assessments reveal significantly decreased locomotor behavior in null mice compared with WT. (**C**) No significant differences were observed between WT and *C9orf72*$^{-/-}$ by Rotarod testing. (**D**) Longitudinal body weights recorded as the change in weight in grams of WT vs. *C9orf72*$^{-/-}$ shows that beginning at 32 weeks of age, *C9orf72*$^{-/-}$ body weights level off and begin to significantly decrease in comparison to WT. (**A**) 0-3 scale is defined as 0=no symptoms, 1=mild, 2=moderate, and 3=severe. (**A-D**) Clinical neurological exams, motor assessment, gait abnormalities and longitudinal body weights were performed on 32-60 week old WT and *C9orf72*$^{-/-}$ mice (n≥14, grouped males and females). Data are represented as mean ± s.e.m (* P≤.05, ** P≤.01, *** P≤.001 and **** P≤.0001 by unpaired Students *t*-test).

# Supplemental Figure 3

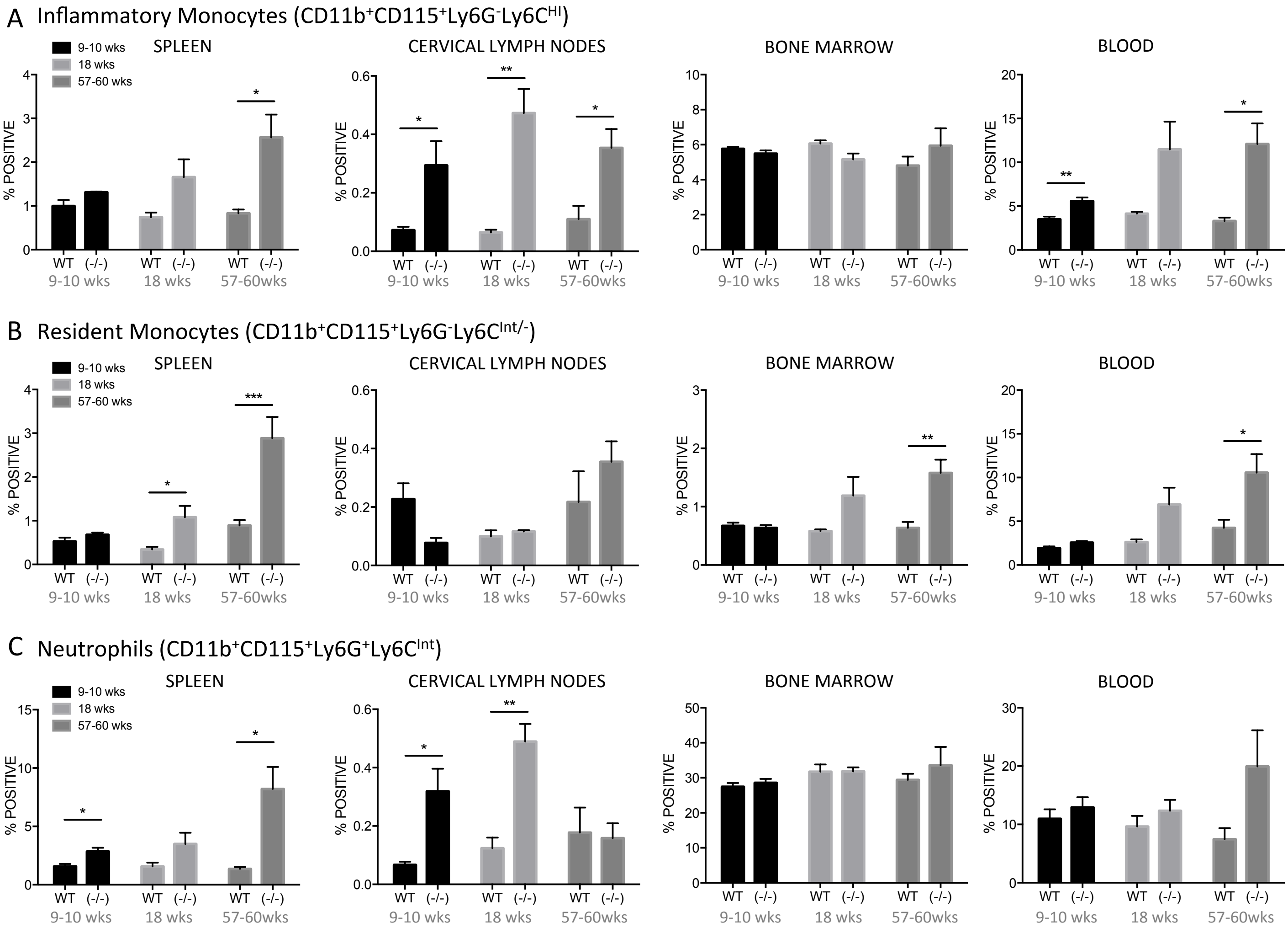


**Supplemental Fig. 3** Male FACS summary data shows increased percentages of myeloid cell populations in spleen, LN, BM, and blood. (**A**) Percent of inflammatory monocytes (CD11b$^+$CD115$^+$Ly6G$^-$Ly6C$^{Hi}$) are increased in spleen, cervical LN, and blood at all time points analyzed with varying significance in null mice. (**B**) Percent of resident monocytes (CD11b$^+$CD115$^+$ Ly6G$^-$ Ly6C$^{Int/-}$) in *C9orf72*$^{-/-}$ are comparable to WT or slightly decreased in all tissues at 9-10 weeks. By 18 weeks, an increase in resident monocytes is observed in all *C9orf72*$^{-/-}$ tissues; this trend continues with varying levels of significance through 57-60 weeks of age. (**C**) Percent of neutrophils (CD11b$^+$CD115$^-$Ly6G$^+$Ly6C$^{Int}$) are increased in *C9orf72*$^{-/-}$ spleen with varying significance at all time points; likewise, an increase in neutrophil percentages is observed in cervical LN through 18 weeks of age with a return to values comparable to WT by 57-60 weeks of age. Neutrophils in BM and blood are demonstrated to be either comparable or slightly increased in *C9orf72*$^{-/-}$ compared with WT. (**A-C**) Data shown represents males aged 9-60 weeks, n=4 per genotype. Graphs represent mean ± s.e.m. (* P≤.05, ** P≤.01 and *** P≤.001 by unpaired Students *t*-test).

# Supplemental Figure 4

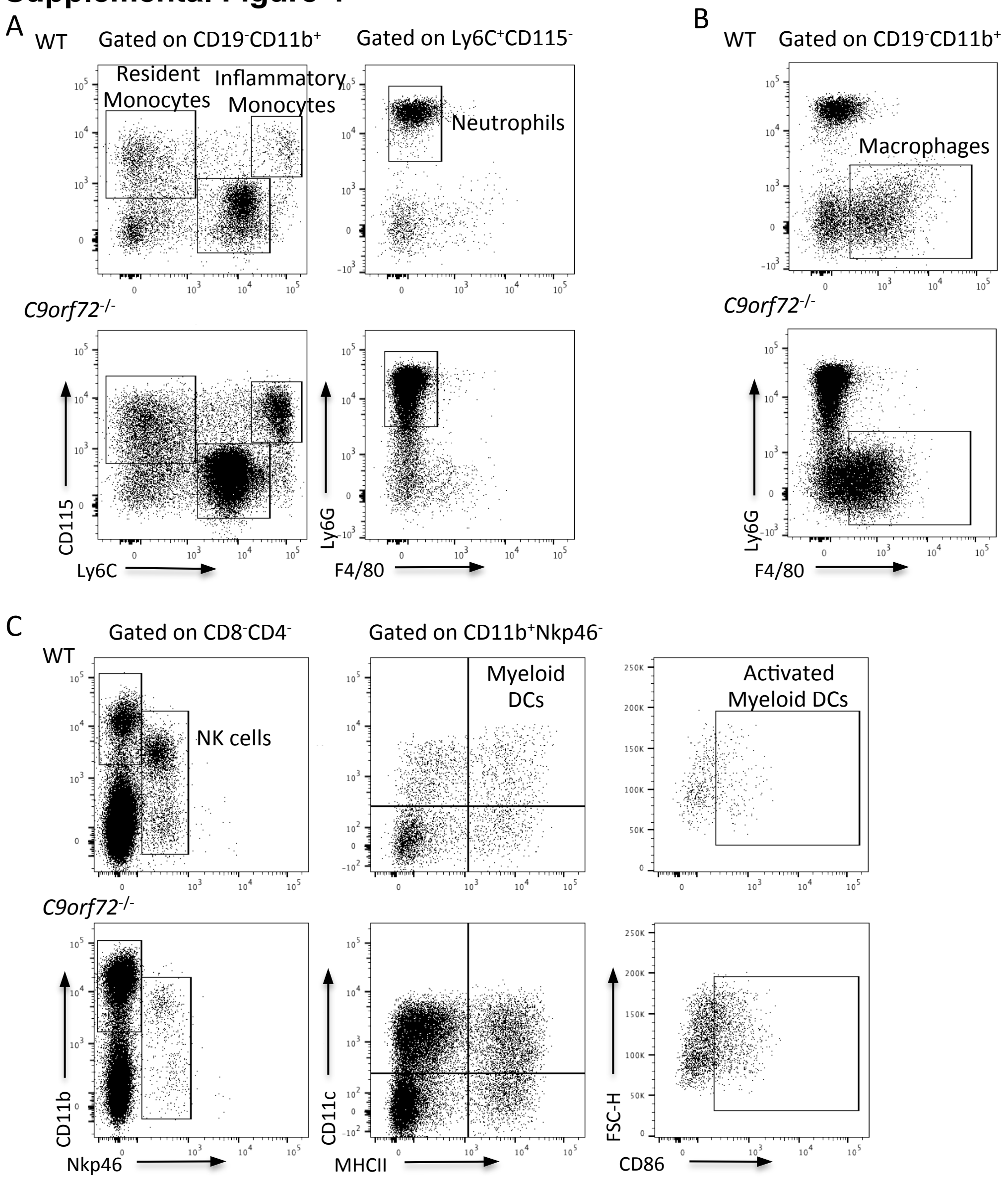


**Supplemental Fig. 4** Representative gating strategy for Myeloid cell populations. Representative FACS plots show WT and *C9orf72*$^{-/-}$ spleen from 26-35 week old females.

## Supplemental Figure 5

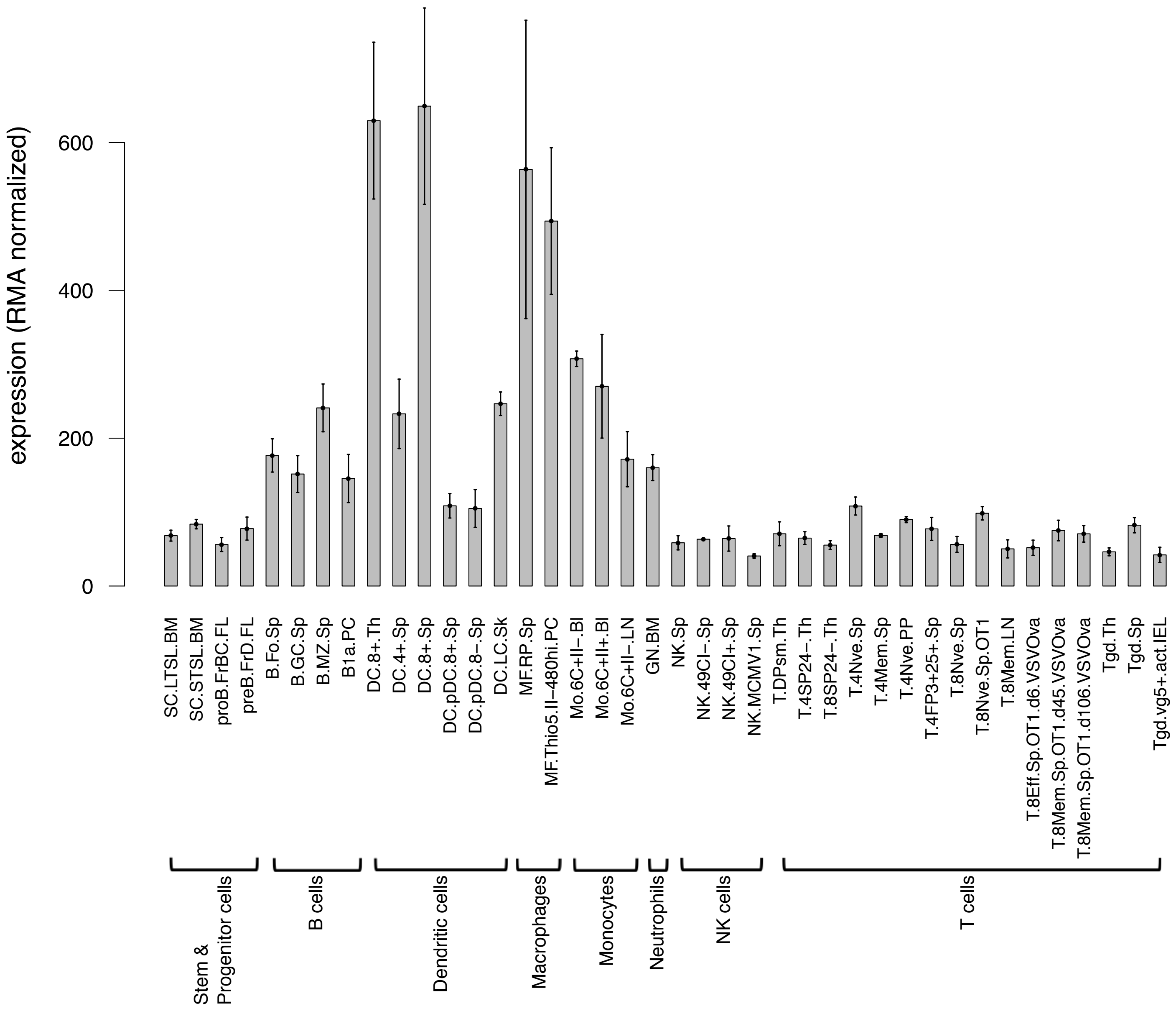


**Supplemental Fig. 5** RMA normalized expression levels for *C9orf72* across immune cell types. Data was obtained from The Immunological Genome Project (ImmGen), a public resource that utilizes microarray data generated from a stringent expression-profiling pipeline. Among immune cells, expression of *C9orf72* is prominent in monocyte, macrophage and dendritic cell populations, with lower expression observed in lymphocytes corresponding to affected cell populations observed in our *C9orf72* deficient mouse line.

## Supplemental Figure 6

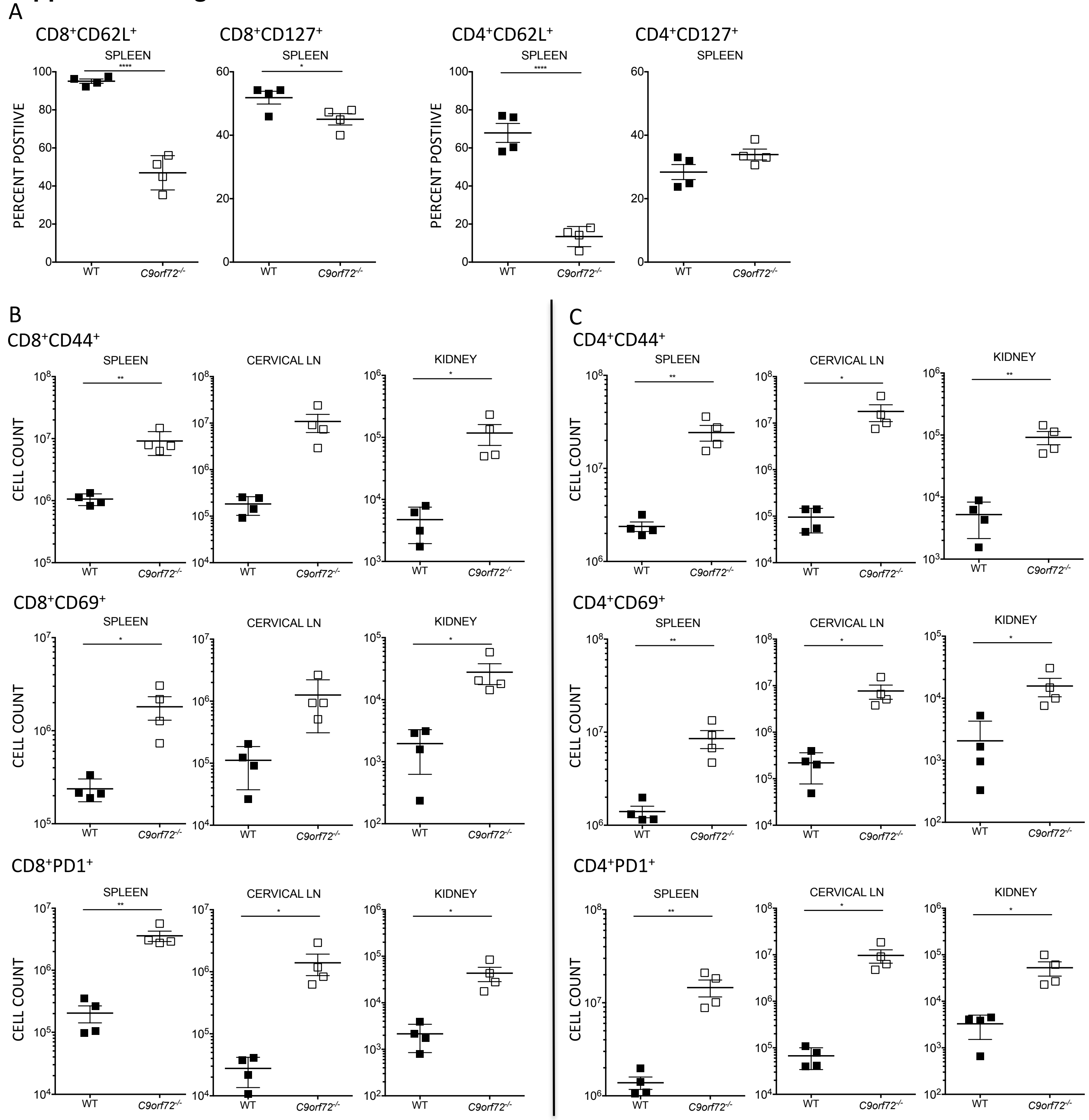


**Supplemental Fig. 6** *C9orf72-/-* mice display decreased percentages of naïve and memory T cell markers and an increased number of activated T-cells. (**A**) Decreased expression of CD62L and CD127 in *C9orf72-/-* CD8+ T cells further confirms T cell activation. CD62L is also decreased in *C9orf72-/-* CD4+ T cells while CD127 is relatively unchanged. (**B,C**) Corresponding to increased percentages indicated in Figure 4, CD8+ and CD4+ T cell summary data reflect increases in the absolute number of *C9orf72-/-* cytotoxic and helper T cells expressing CD44, CD69, and PD1 in comparison to WT. Graphs represent mean ± s.e.m. (* P≤.05, ** P≤.01 and *** P≤.001 by unpaired Students *t*-test) 30-35 week females, n=4 per genotype.

## Supplemental Figure 7

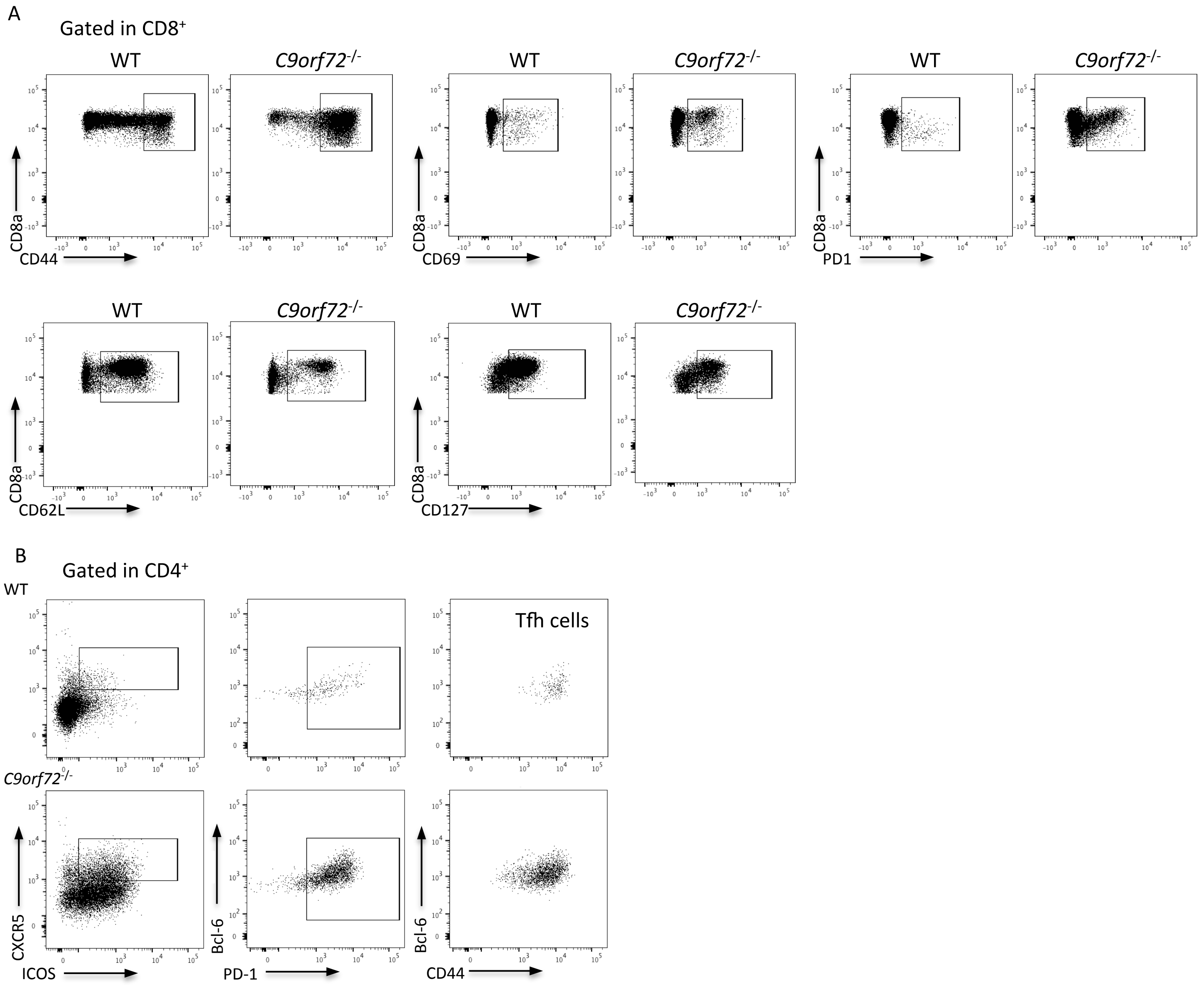


**Supplemental Fig. 7** Representative gating strategy for T cell populations. (**A**) FACS plots are shown for activated $CD8^+$ T cell populations in 30-35 week old female WT and *C9orf72*$^{-/-}$ spleen. Similar gating strategy was applied to $CD4^+$ activated T cell populations (**B)** FACS gating strategy for Tfh cells ($CD4^+CXCR5^+CD44^+ICOS^+PD\text{-}1^+Bcl\text{-}6^+$). Representative FACS plots show WT and *C9orf72*$^{-/-}$ spleen from 26 week old females.

## Supplemental Figure 8

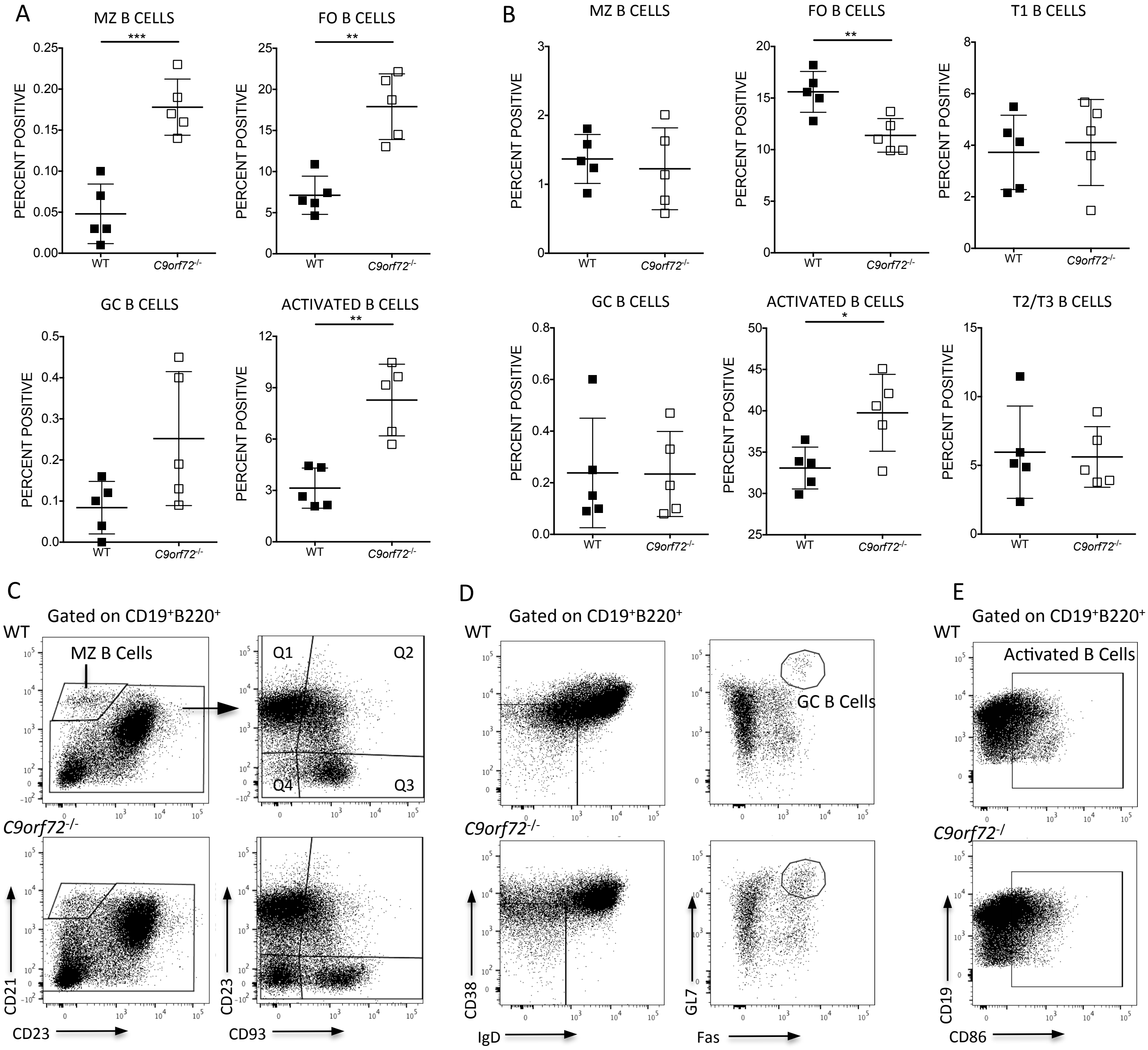


**Supplemental Fig. 8** B cell populations in *C9orf72*$^{-/-}$ spleen and cervical LN with representative FACS plots (**A**) FACS analysis using markers for specific B cell subsets reveal increased MZ, F0, GC and activated B cell populations in *C9orf72*$^{-/-}$ cervical LN compared with WT. (**B**) FACS analysis using markers for specific B cell subsets reveal increased activated B cell populations in *C9orf72*$^{-/-}$ spleen. MZ, F0, GC, T1, T1/T2, subsets are reduced or relatively unchanged in C9orf72-/- compared with WT. (**C-E**) Representative FACS plots of WT (top row) and *C9orf72*$^{-/-}$ (bottom row) spleen for (**C**) MZ B cells in the first plot defined as $CD45^+CD19^+B220^+CD21^+CD23^-$ cell population. FO B cells are quantified in the second plot in quadrant 4 ($CD45^+CD19^+B220^+CD21^{+/-}CD23^+CD93^-$); T1 cells in quadrant 3 ($CD45^+CD19^+B220^+CD21^-CD23^-CD93^+$); and T2/T3 cells in quadrant 2 ($CD45^+CD19^+B220^+CD21^{+/-}CD23^+CD93^+$). (**D**) GC B cells are $CD45^+CD19^+B220^+CD38^-IgD^-GL7^+Fas^+$ (**E**) Activated B cells are represented by $CD45^+CD19^+B220^+CD86^+$ cell population. (**A,B**) Graphs represent mean ± s.e.m. (* P≤.05, ** P≤.01 and *** P≤.001 by unpaired Students *t*-test) 26week females, n=5 per genotype.

## Supplemental Figure 9

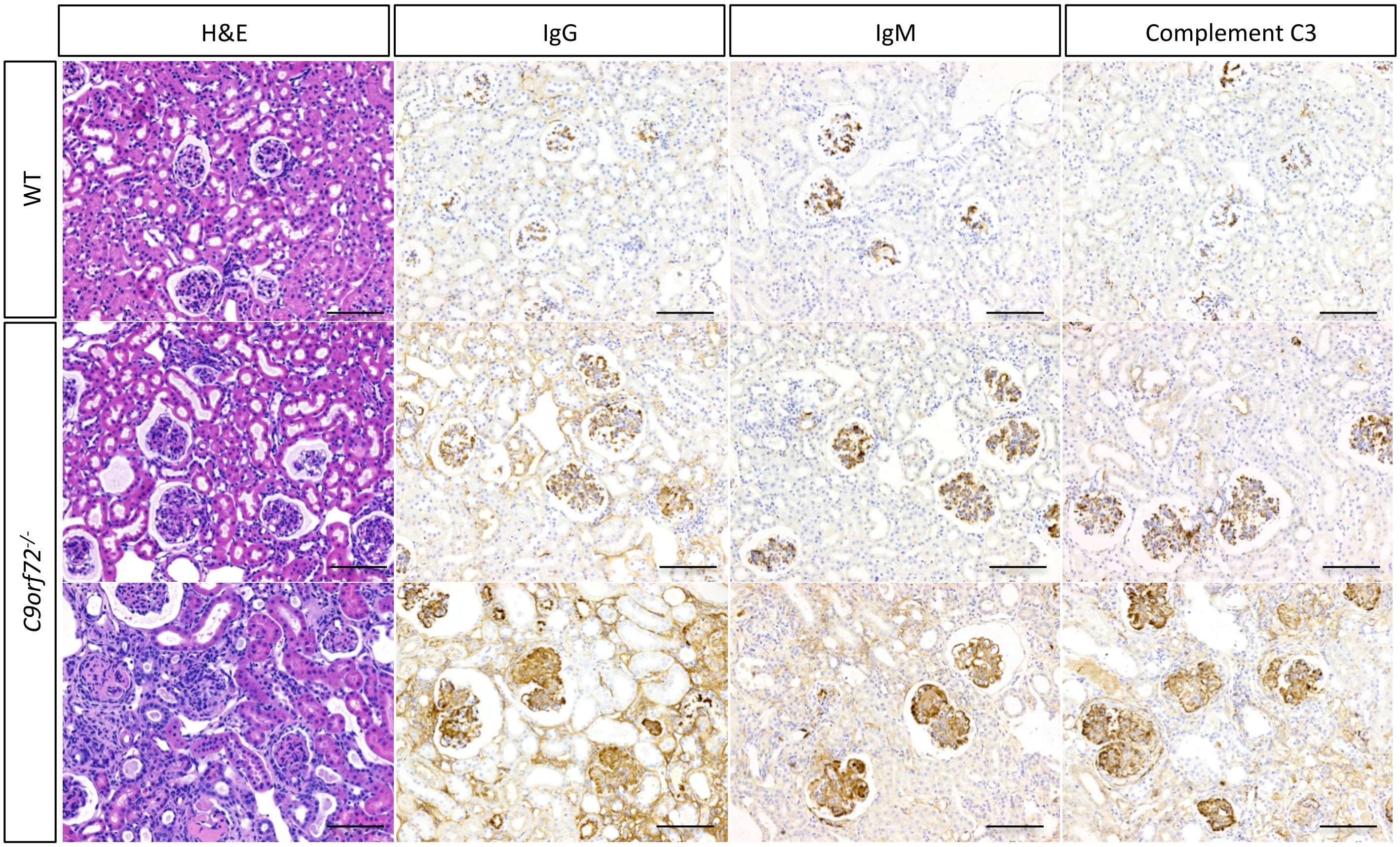


**Supplemental Fig. 9** Increased IgG, IgM and Complement C3 by IHC in *C9orf72*$^{-/-}$ kidney is characteristic of immune-mediated kidney damage. WT renal cortex (top row) compared with *C9orf72*$^{-/-}$ rental cortex (middle and bottom rows) indicates that null mice present with mild (middle row) and marked (bottom row) proliferative glomerulonephropathy. As described, H&E staining demonstrates progressive changes, from mild to marked, in immune-mediated disease in *C9orf72*$^{-/-}$ kidney. Immunoreactivity to IgG, IgM, and C3 likewise increases with disease severity within the glomeruli of null mice compared with WT. Marked increase in staining is also observed within the adjacent renal parenchyma, particularly of proximal tubules, indicating permissive leak of glomerular basement membranes of null mice to the lower molecular weight IgG (2$^{nd}$ column). IgM is largely confined to the glomerulus (column 3). C3 immunoreactivity is present on capillary loops (column 4). Data represented is from 60-63 week old females, n=5 per genotype analyzed. Scale bar represents 100 µm, original magnification, x200.

## Supplemental Table 1

| Genotype | Age (wks) | Membranoproliferative Glomerulonephritis | Interstitial Mononuclear Inflammation | Hyaline Cast | Glomerulosclerosis | Basophilic Tubules |
|---|---|---|---|---|---|---|
| WT | 38 | 0 | 0 | 0 | 0 | 0 |
| WT | 38 | 0 | 0 | 0 | 0 | 0 |
| WT | 40 | 0 | 0 | 0 | 0 | 0 |
| WT | 63 | 0 | 0 | 1 | 0 | 0 |
| WT | 63 | 0 | 0 | 0 | 0 | 0 |
| WT | 63 | 0 | 0 | 1 | 0 | 0 |
| WT | 63 | 0 | 0 | 0 | 0 | 0 |
| WT | 61 | 0 | 0 | 1 | 0 | 0 |
| *C9orf72*$^{-/-}$ | 38 | 1 | 0 | 0 | 0 | 0 |
| *C9orf72*$^{-/-}$ | 38 | 2 | 0 | 0 | 0 | 1 |
| *C9orf72*$^{-/-}$ | 37 | 4 | 2 | 3 | 2 | 2 |
| *C9orf72*$^{-/-}$ | 37 | 1 | 0 | 0 | 0 | 0 |
| *C9orf72*$^{-/-}$ | 37 | 3 | 3 | 3 | 1 | 3 |
| *C9orf72*$^{-/-}$ | 35 | 1 | 0 | 0 | 0 | 0 |
| *C9orf72*$^{-/-}$ | 63 | 1 | 0 | 1 | 0 | 0 |
| *C9orf72*$^{-/-}$ | 63 | 2 | 0 | 0 | 0 | 0 |
| *C9orf72*$^{-/-}$ | 61 | 3 | 3 | 3 | 3 | 0 |
| *C9orf72*$^{-/-}$ | 61 | 2 | 0 | 1 | 0 | 1 |
| *C9orf72*$^{-/-}$ | 61 | 3 | 0 | 1 | 0 | 0 |

**Supplemental Table 1** Individual histopathological scores of aging *C9orf72*$^{-/-}$ kidney and WT controls. H&E stained kidney sections were blindly scored for membranoproliferative glomerulonephritis, interstitial mononuclear inflammation, hyaline cast formation, glomerulosclerosis, and basophilic tubules; categories of renal disease associated with immune mediated glomerulonephropathy. Score of 0=none, 1=minimal, 2=mild, 3=moderate and 4=severe. All null mice display minimal to severe membranoproliferative glomerulonephritis with occasional evidence of additional disease categories in more severely affected animals.
